# Interventions that support women, girls, and people who menstruate to participate in physical activity: a rapid overview of reviews

**DOI:** 10.1186/s12889-026-27122-9

**Published:** 2026-03-27

**Authors:** Judit Csontos, Natalie Brown, Adrian Edwards, Deborah Edwards, Elizabeth Gillen, Juliet Hounsome, Meg Kiseleva, Ruth Lewis, Steven Macey, Mala Mann, Amrita Sidhu, Alison Cooper

**Affiliations:** 1 Wales Centre For Evidenced Based Care (WCEBC), Cardiff University, Cardiff, Wales, UK; 2 School of Healthcare Sciences, Cardiff University, Cardiff, UK; 3 Welsh Institute of Performance Science, Swansea University, Swansea, UK; 4 Sport and Exercise Science, Swansea University, Swansea, UK; 5 Division of Population Medicine, School of Medicine, Cardiff University, Cardiff, UK; 6 Health and Care Research Wales Evidence Centre, Cardiff University, Cardiff, UK; 7 Specialist Unit for Review Evidence (SURE), Cardiff University, Cardiff, UK; 8 North Wales Centre for Primary Care Research, Bangor University, Wrexham, UK; 9 Equality, Poverty and Children’s Evidence and Support Division, Welsh Government, Swansea, UK

**Keywords:** Physical activity, Exercise, Sport, Menstruation, Menstrual cycle, Women’s health, Children, Adolescents, Rapid review, Evidence synthesis

## Abstract

**Background:**

Adults (age 18–64 years) are recommended 150–300 min of moderate-to-vigorous or 75–150 min of vigorous-intensity physical activity per week. Irrespective, the number of women not meeting recommended physical activity is 5% higher than men globally. Women, girls and people who menstruate face multiple barriers to physical activity participation, including gender bias, low perceived exercise competence, and insufficient support from peers and/or family. Moreover, menstruation is often reported as a barrier. Numerous interventions have been proposed to increase women’s and girls’ physical activity participation, while little is known about interventions for people who menstruate. Therefore, the aim of this rapid overview of reviews was to summarise systematic reviews investigating the effectiveness of interventions that support women, girls, and people who menstruate to participate in physical activity. Additionally, this review examined whether any of these interventions incorporated managing physical activity participation throughout the menstrual cycle.

**Methods:**

Bibliographic databases (MEDLINE, Emcare, and AMED on OVID platform; CINAHL and SPORTDiscus on EBSCO; Epistemonikos, and Cochrane Database) and grey literature were searched in March 2024. Title/abstract screening was conducted by one reviewer and 20% of records checked by a second. Full-text screening was performed by two reviewers. Data extraction and critical appraisal (using JBI systematic review checklist) were conducted by one reviewer with another checking accuracy. Findings were summarised narratively.

**Results:**

Fifteen systematic reviews across 16 reports (published between 2008 and 2024) met the inclusion criteria. The population included young and adolescent girls (*n* = 9), adult women (*n* = 3), mothers and daughters (*n* = 2), and mixed population (*n* = 1). A wide range of interventions were identified which were educational, environmental or multicomponent. None of the included systematic reviews described interventions focusing on managing physical activity participation throughout the menstrual cycle. Most reviews found mixed effectiveness in increasing physical activity participation, leading to inconclusive results.

**Conclusion:**

While multicomponent interventions may lead to better physical activity outcomes, it is unclear what combined elements are the most effective. There is a need for high quality research to identify interventions that could help girls, women, and people who menstruate to be physically active, and consider barriers related to the menstrual cycle.

**Review registration:**

https://doi.org/10.17605/OSF.IO/XTYCW

**Supplementary Information:**

The online version contains supplementary material available at 10.1186/s12889-026-27122-9.

## Background

Insufficient physical activity is a significant risk factor for non-communicable diseases alongside having a negative impact on mental health and quality of life [1]. Hence, it is important that people maintain adequate physical activity levels throughout their lifetimes. The World Health Organization (WHO) provides evidence-based recommendations on required activity levels for children and adolescents (5–17 years), adults (18–64 years), older adults (65 years and older), pregnant and postpartum women, adults with chronic conditions, and for disabled children, adolescents and adults [2]. Children and adolescents should perform moderate- to vigorous-intensity physical activity (MVPA) at least 60 min per day, and vigorous-intensity aerobic and strengthening exercises at least three times a week [2]. Adults between the age of 18 and 64 years are recommended either 150 to 300 min of MVPA or 75 to 150 min of vigorous-intensity physical activity per week [2]. However, the prevalence of insufficient physical activity (less than 150 min/week moderate-intensity, or 75 min/week vigorous-intensity exercise, or any combination of these) is 5% higher among women than in men globally [1]. In the United Kingdom (UK), while the Department of Health & Social Care [3] adopted physical activity guidelines similar to the WHO, women (16 years and older) were also less likely to be physically active (61%) compared to men (65.6%) [4]. A similar trend also exists in Wales, where women were more likely to report ‘no activity’ (18%) compared to all adults (16%) [5].

The lower level of physical activity participation of women is particularly important, as evidence suggests that women experience more ill health and impairments throughout the life course [6]. Meanwhile, the benefits of being physically active are well documented [2], particularly regarding leisure-domain physical activity because it has a stronger association with improved mental and physical health compared to other domains, such as work or household [7, 8]. However, participation in leisure-domain physical activity is 3.1% lower in women than in men globally [9]. Thus, it is important to understand how to support women to participate in leisure-domain physical activity. Since the decline in physical activity often starts in puberty [10–12], it is also crucial to understand adolescent girls’ experiences and investigate what could support them to be physically active during their formative years to help them become active adults [11].

Several barriers to the physical activity participation of women and girls have been documented in the literature. Adolescent girls experience gender bias, low perceived exercise skills and competence, lack of support from peers and/or family to be physically active, and have limited time and competing priorities [13, 14]. Women between the ages of 18 and 87 years often reported barriers, such as body image and societal beauty standards, family duties, lack of social support, religious and cultural norms, inadequate sport facilities and environment, and safety issues [15]. Transgender men and non-binary individuals, referred to as people who menstruate from this point forward [16], can face barriers related to discrimination, the physical environment, for example changing rooms, and fear their transition will be exposed [17]. Physiological differences between sexes can also act as a barrier, for example, menstruation and symptoms associated with periods are often reported as factors that reduce physical activity among adults and adolescents [18–20]. In the UK, a recent survey exploring adolescent girls’ physical activity behaviour found that 84% reported reduced interest in sport and physical activity following menarche and 23% felt embarrassed to participate in exercise during their periods [10]. Other reports suggest that seven in 10 young and adolescent girls avoid physical activity during their periods [12]. Beside feelings of embarrassment related to periods, other issues associated with the menstrual cycle that are reported to reduce physical activity include pain, discomfort, low mood and energy, fear of leaking, and feeling self-conscious in changing rooms [20, 21]. When these barriers go unaddressed, they can also become inhibiting factors in adulthood with women often reporting periods as a reason for lack of physical activity [10, 19, 22, 23].

The barriers affecting women and girls have become recognised worldwide, and in recent years, there has been increased policy focus on getting women, girls, and people who menstruate active, with recommendations being made to overcome inhibiting factors. The Office on Women’s Health, USA, has dedicated pages to promoting physical activity with information provided on staying safe and the importance of keeping active during menstruation [24]. In Australia, the ‘National Women’s Health Strategy 2020–2030’ prioritised the promotion of physical activity, with recommendations made for tailored interventions, and improving healthcare professionals’ exercise literacy [25]. The House of Commons [26], UK, and the Scottish Parliament [27] made recommendations, such as improved education regarding the menstrual cycle and change in physical education kit to address barriers affecting physical activity participation of adolescent girls. Additionally, the Period Proud Action Plan in Wales, UK, have set out to gather evidence and make recommendations on interventions that could support physical activity participation of women, girls, and people who menstruate, particularly solutions to increase activity levels throughout the menstrual cycle [28].

Numerous interventions have been evaluated throughout the years to increase the physical activity participation of women and girls. School- or community-based interventions have been investigated for young and adolescent girls [29], while workplace interventions have been suggested for adult women [30, 31]. Preliminary searches identified multiple systematic reviews focusing on interventions that aim to support physical activity participation of women, girls and people who menstruate [32]. Therefore, the aim of this rapid overview of reviews was to summarise systematic reviews investigating the effectiveness of interventions that support women, girls, and people who menstruate to participate in physical activity. The focus was mainly on leisure-domain physical activity, including exercise and sport. As this rapid overview of reviews was intended to inform the Period Proud Action Plan, this work also examined whether the included systematic reviews contained interventions that could help manage physical activity participation throughout the menstrual cycle.

## Methods

Overviews of reviews (or umbrella reviews) are a type of evidence synthesis that can summarise evidence from multiple systematic reviews [33, 34]. They are useful in broad topic areas where numerous systematic reviews have already been undertaken and can provide opportunity for bringing together evidence from different interventions or populations ensuring an account of totality [33, 34]. Overviews of reviews can also help reduce research waste by using information from already existing systematic reviews [33]. Therefore, this methodology suited this review which aimed to bring together evidence from multiple systematic reviews focusing on a wide range of interventions that support women, girls and people who menstruate to participate in physical activity. Rapid review methodology was also used for this project to meet the timelines of a local stakeholder group who were gathering evidence to inform the Period Proud Action Plan focusing on physical activity participation of women, girls and people who menstruate [28]. Rapid overview of reviews is an established evidence synthesis methodology, commonly used in policy making and rapid research, when limited time is available [35–37].

This rapid overview of reviews was based on Cochrane rapid review guidance and the synthesis of systematic review evidence was informed by JBI umbrella review methodology [33, 38]. A protocol detailing the methods was developed prior to the start of this rapid overview of reviews, and registered on Open Science Framework (OSF) [39]. The conduct of this review was reported according to the Preferred Reporting Items for Systematic reviews and Meta-Analyses (PRISMA) 2020 [40] and the Reporting Items for Overviews of Reviews (PRIOR) [41]. The reporting checklists are presented in additional files 1 and 2.

### Eligibility criteria

The Population, Intervention, Comparator, Outcome (PICO) framework was used to inform the eligibility criteria (Table 1). This rapid overview of reviews included systematic reviews that focused on interventions that support women, girls, and people who menstruate to participate in physical activity, with specific focus on life stages when this population can have a menstrual cycle. These life stages were chosen as menstruation can often act as a barrier to physical activity [21], thus it is crucial to examine whether interventions consider this issue. This population was also of interest to stakeholders working on the Period Proud Action Plan. Women, girls, and people who menstruate that experience menstruation disturbances were also included, as dysmenorrhea and heavy bleeding could present further obstacles to being physically active. However, menopausal, post-menopausal and pregnant women were excluded as these populations do not have an active menstrual cycle and due to their circumstances may need alternative interventions that is more specific to their needs [42–44]. These circumstances may include sleeping difficulties and fatigue in menopausal women [42], or pregnancy discomforts [44]. Additionally, postpartum women were also excluded as their distinct needs regarding potential recovery and breastfeeding may require additional support [43]. Systematic reviews solely focusing on populations with obesity, poor nutrition, or those who smoke, and people living with chronic health conditions, such as diabetes, cardiovascular disease or cancer were also excluded, as these conditions may require more specialist input involving healthcare services.

**Table 1:** Eligibility criteria

	Inclusion criteria	Exclusion criteria
Population	Women, girls, and people who menstruate from menarche^a^ up to menopause^b^; Women, girls, and people who menstruate with menstruation disturbances and associated conditions	Girls prior to menarche; Menopausal and post-menopausal women; Women, girls, and people who menstruate participating and competing in sports at an elite level; Pregnancy; Postpartum women^c^; Women, girls, and people who menstruate with a chronic health condition (e.g. cancer, diabetes, cardiovascular disease, etc.) or obesity, smoking, and poor nutrition
Intervention / exposure	Interventions that support women, girls, people who menstruate to participate in leisure-domain physical activity^d^ / exercise^e^ / sport^f^; Interventions that manage leisure-domain physical activity^d^ / exercise^e^ / sport^f^ participation throughout the menstrual cycle	Interventions focusing on behaviour change of people with a chronic health condition (e.g. cancer, diabetes, cardiovascular disease, etc.) or obesity, poor nutrition, and smoking (e.g. weight management interventions, smoking cessation); Exercise-based prehabilitation / rehabilitation for surgical interventions or treatments for health conditions; Enhanced Recovery After Surgery (ERAS)
Comparator	Any	
Outcome	Participation; Attendance; Leisure-domain physical activity / exercise / sport behaviour; Leisure-domain physical activity / exercise / sport frequency, intensity, or duration	
Setting / Context	Any	
Study design	Quantitative systematic reviews; Mixed methods systematic reviews using segregated approach to synthesize the findings	Primary studies; Narrative or non-systematic reviews; Scoping reviews; Qualitative systematic reviews; Mixed methods systematic reviews using an integrated approach to synthesize the findings
Language of publication	English	Languages other than English
Publication date	2008 - Current	Reviews published prior to 2008
Publication type	Published and preprint, grey literature	
Other factors *Any other key points to note*	No geographical limitations	

^a^ Menarche: “The start of menstrual periods in adolescence” [45]. “This is usually between age 8 and 17” [46]

^b^ Menopause: “Menopause is a biological stage in a woman's life when menstruation stops permanently due to the loss of ovarian follicular activity. It occurs with the final menstrual period and is usually diagnosed clinically after 12 months of amenorrhoea. In the UK, the mean age of natural menopause is 51 years, although this can vary between different ethnic groups” [47]

^c^ Postpartum: “Postpartum refers to a period of time after the end of pregnancy. The postpartum period is commonly defined as up to six weeks following the end of pregnancy, with the late postpartum period from six weeks up to one year after the end of pregnancy” [3]

^d^ Leisure-domain physical activity: “Physical activity performed by an individual that is not required as an essential activity of daily living and is performed at the discretion of the individual. Such activities include sports participation, exercise conditioning or training, and recreational activities such as going for a walk, dancing, and gardening” [2]

^e^ Exercise: “A subcategory of physical activity that is planned, structured, repetitive, and purposeful in the sense that the improvement or maintenance of one or more components of physical fitness is the objective. “Exercise” and “exercise training” frequently are used interchangeably and generally refer to physical activity performed during leisure time with the primary purpose of improving or maintaining physical fitness, physical performance, or health” [2]

^f^ Sport: “Sport covers a range of activities performed within a set of rules and undertaken as part of leisure or competition. Sporting activities involve physical activity carried out by teams or individuals and may be supported by an institutional framework, such as a sporting agency” [2]

Any intervention aiming to increase leisure-domain physical activity, exercise or sport participation were considered for inclusion. The systematic reviews reporting any physical activity outcomes were considered for inclusion, encompassing subjective methods, such as self-report via physical activity questionnaires, diaries or logs, and objective measures, for example accelerometers, pedometers, or heart rate monitors [48].

### Searching

A comprehensive search of bibliographic databases was conducted for English language publications from 2008 to March 2024. The date limit of 2008 was applied, as an evidence review by The National Institute for Health and Care Excellence (NICE) [29] focusing on physical activity promotion for adolescent girls was published that year, which was a comprehensive exploration of previously published randomised controlled trials. The following bibliographic databases were searched: Medline, Emcare, and AMED on OVID platform; CINAHL and SPORTDiscus on EBSCO; Epistemonikos; and Cochrane Database of Systematic Reviews. The search strategy was developed by an experienced information specialist (EG) and tailored for each information source (see additional file 3). The websites of key third sector and government organisations were also searched (see additional file 4). Forward and backward citation searching was completed using Web of Science and relevant studies were added to the review.

### Screening

All citations retrieved from the database searches were imported into EndNote™ (Thomson Reuters, CA, USA) and duplicates were removed. Following deduplication, the remaining citations were imported to Rayyan™, where two reviewers (JC, AS) dual screened approximately 20% of citations using the information provided in the title and abstract. The rest of the citations were screened by a single reviewer (JC). Any conflicts in the title and abstract screening were resolved by a third reviewer (EG). For citations that appeared to meet the inclusion criteria, or in cases in which a definite decision could not be made based on the title and abstract alone, the full texts were retrieved. The full texts were screened for inclusion by two reviewers (JC, EG, JH, MK, MM, AS) and any disagreements were resolved by a third reviewer (JC, EG). Systematic reviews that contained a wider population range than the inclusion criteria, for example a review that included studies with young and post-menopausal women, were included if the majority (75%) of the review population met the inclusion criteria [49]. To minimise the chance of missing key information, in addition to checking the full-texts, included studies tables within the systematic reviews, their data extraction tables in available additional materials, and titles of included primary research studies within the systematic reviews were thoroughly examined.

### Assessment of methodological quality

Eligible systematic reviews were critically appraised using the JBI critical appraisal checklist for systematic reviews and research syntheses [33]. Methodological quality assessment was conducted by one reviewer (JC, DE, EG, JH, MK, MM, AS) and checked by a second (DE, MK, MM) and any disagreements were resolved by a third researcher (JC, DE). All included systematic reviews regardless of the results of their methodological quality, underwent data extraction and synthesis.

### Data extraction and assessment of the body of evidence

Data were extracted directly into a data extraction table by one reviewer (JC, DE, EG, JH, MK, MM, AS) and checked by another (JC, DE). The data extracted included specific details about the included systematic reviews (purpose of the review; number, publication date, research design and quality rating of included studies; populations; interventions (type, length, setting and country); and outcomes of significance to the review questions and objectives). Data extraction process was piloted on one report to see whether the data extraction table was fit for purpose.

To investigate whether any of the included systematic reviews contained interventions that aimed to support physical activity participation throughout the menstrual cycle, full-text documents, included studies tables, and additional materials were thoroughly examined. If information was provided on any interventions focusing on managing the menstrual cycle, it was extracted. Additionally, titles of all included studies within the systematic reviews were tabulated and examined for any mention of menstruation, period or menstrual cycle.

To determine the certainty in the evidence, existing Grading of Recommendations Assessment, Development and Evaluation (GRADE) results were also extracted from the included systematic reviews [50]. For systematic reviews where GRADE assessment had not been completed, the GRADE assessment checklist developed by Meader et al. [51] was used to try to determine the certainty in the evidence [34].

### Synthesis

The data extracted from selected reviews was tabulated and reported narratively as a series of thematic summaries [52]. Results were organised based on the population, focusing on different age categories or specific groups, such as mothers and daughters, to ensure their data can be considered separately. Where systematic review authors conducted a meta-analysis, effect estimates (for example standardized mean difference), and data on heterogeneity (I^2^, Cochran Q, or τ^2^) were presented in a tabular format. No further meta-analysis or re-analysis of the data was performed as recommended by JBI [33].

As this work was a rapid overview of reviews, it is possible that identified systematic reviews include the same primary research studies. This phenomenon is called overlap, which could lead to overestimation of results [52]. To determine the degree of overlap, the corrected covered area was calculated [66]. Based on the corrected covered area, less than 5% overlap is a slight overlap, 6–10% is a moderate overlap, 11–15% is a high overlap and > 15% is a very high overlap [66].

## Results

### Flow of studies through the review

**Fig. 1:**
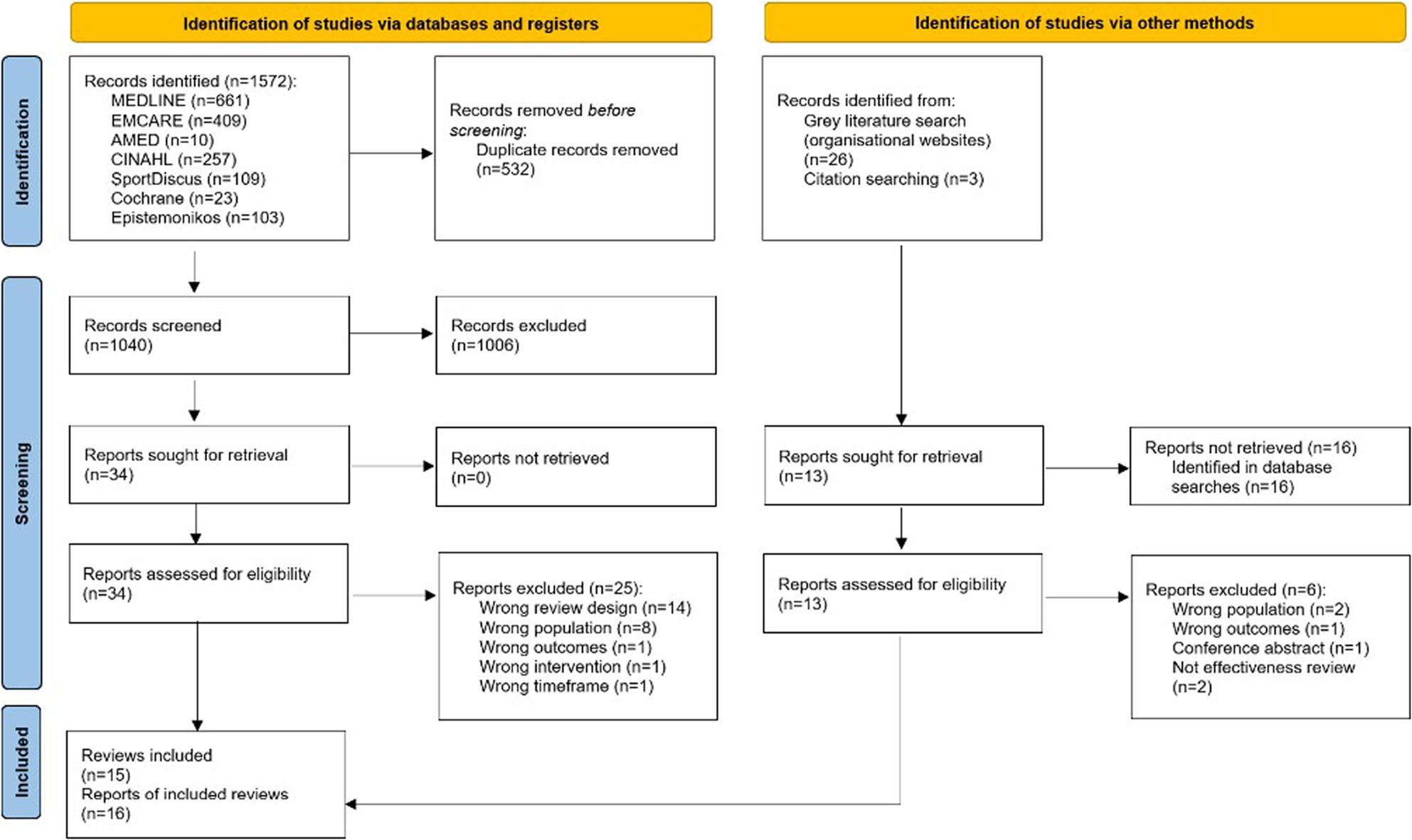
PRISMA flow diagram

The flow of citations through each stage of the review process is displayed in a PRISMA flowchart [40] and can be found in Fig. 1. The database searches identified 1752 records and after duplicates were removed 1040 records underwent title and abstract screening. Following this process, the full-texts of 34 reports were screened against the inclusion criteria. An additional 29 reports were identified from grey literature and citation searching and of these 13 full-texts were screened against the inclusion criteria. Fifteen systematic reviews published across 16 reports were included in this review. The full details of reports excluded at full text screening can be found in additional file 5.

### Characteristics of included reviews

The characteristics of the included reviews can be found in Table 2 and additional file 6 and are also described narratively below.

The included systematic reviews were published between 2008 and 2024. Nine systematic reviews were conducted with young and adolescent girls and interventions were delivered in a variety of settings, including schools (extracurricular / after school activities), communities including clubs and teams, primary care services, at participants’ home, or online [29, 53–56–61]. Three systematic reviews focused on adult women, with interventions delivered in the community or workplace [30, 31, 62]. Two systematic reviews summarised interventions for mothers and daughters, which were conducted in settings, such as the community, after school clubs, home or online [64, 65]. One systematic review included studies conducted with adolescents or adult women [63]. None of the included systematic reviews contained information about interventions for people who menstruate. Information about the ethnicity of study participants was only reported across two of the included systematic reviews [56, 61], with both including African American and Latinx populations.

**Table 2:** Included systematic review characteristics

Citation Number of included studies	Participants Age	Type of interventions: definition/description	Outcome	Impact	GRADE
Young and adolescents girls					
Allison et al. 2017 [53] *n* = 4	Girls 11–25	Partnership working (*n* = 1) Grant funding (*n* = 1) Multicomponent (*n* = 2)	Team sport participation	Mixed (no inferential statistics provided)	NR
Biddle et al. 2014^m^ [54, 55] *n* = 22	Preadolescents 5–12	Educational (*n* = 9) Environmental (*n* = 4) Multicomponent (*n* = 9)	PA participation	*Overall effect* Significant small effect (k = 22, g = 0.314, 95% CI (0.112, 0.516), *p* < 0.001) Heterogeneity: (Q_T_=346.37, *p* < 0.001, τ^2^ = 0.199, I^2^ = 93.94) *Educational* Significant small effect (k = 9, g = 0.414, 95% CI (0.070, 0.759), *p* < 0.01) Heterogeneity: (τ^2^ = 0.067, I^2^ = 82.43) *Environmental* No significant effect (k = 4, g= -0.301, 95% CI (− 0.795, 0.194), *p* > 0.01) Heterogeneity: (τ^2^ = 1.174, I^2^ = 98.11) *Multicomponent* Significant small effect (k = 9, g = 0.503, 95% CI (0.172, 0.833), *p* < 0.01) Heterogeneity: (τ2 = 0.174, I^2^ = 93.17)	NR
Camacho-Minano et al. 2011 [56] *n* = 21 studies across 29 reports	Young and adolescent girls 5–18	Educational and multicomponent but interventions are not formally grouped	PA levels/ PA behaviour	Mixed (Positive effect (*n* = 10/21))	NR
Houle et al. 2020 [57] *n* = 17 (4 reported on PA outcomes and are extracted)	Adolescent girls 11–17	Extra-curricular physical activity programs PA programme (*n* = 2) Multi-approach programme (*n* = 2)	Mean daily MVPA minutes	No effect	NR
Kelly et al. 2024 [58] Peer reviewed paper (*n* = 1) Grey literature (*n* = 15)	Peer reviewed paper: Adolescents 11–16 Grey literature: Not reported	Peer reviewed paper: an elite sports role model visited the schools Grey literature: Sport role model encounters. One off encounters (*n* = 10), multiple encounters (*n* = 5, 2 with minimal interaction)	PE attendance	No effect (Peer-reviewed paper) Unavailable/unpublished evaluations (grey literature)	NR
NICE 2008 [29] *n* = 12 (13 intervention arms)	Adolescent girls 11–18	Counselling (*n* = 1) Mediated (*n* = 4) Mediated & Counselling (*n* = 2) Education (*n* = 4) PA self-monitoring (*n* = 1) Education & Mediated (*n* = 1)	PA outcomes	*Mediated interventions* Mixed (Positive effect (*n* = 2/6)) *Counselling interventions* Mixed (Positive effect (*n* = 1/3)) *Educational interventions* Mixed (Positive effect (*n* = 3/5))	NR
Owen et al. 2017^m^ [59] *n* = 20	Adolescent girls 11–17	Multicomponent (*n* = 10) Single component interventions (*n* = 10)	PA outcomes	*Overall effect* Significant small effect (k = 17, g = 0.37, 95% CI (0.0008, 0.73), *p* < 0.05) Heterogeneity: (Q = 80.12, *p* < 0.001; I^2^ = 94.91%) *Overall effect (without outlier)* No significant effect (k = 16, g = 0.07, 95% CI (− 0.002, 0.14), *p* = 0.05) Heterogeneity: (Q = 23.98, *p* > 0.05; I^2^ = 0.01%) *Single component intervention* No significant effect (k = 9, g = 0.02, 95% CI (− 0.09, 0.14), *p* > 0.05) Heterogeneity: (Q = 11.83, *p* > 0.05; I^2^= 0.00%) *Multicomponent intervention* Small significant effect (k = 7, g = 0.09, 95% CI (0.006, 0.18), *p* < 0.05) Heterogeneity: (Q = 11.30, *p* > 0.05; I^2^= 0.02%)	NR
Pearson et al. 2015^m^ [60] *n* = 34 studies (independent samples) across 45 reports	Adolescent girls 12–18	Educational (*n* = 21) Environmental (*n* = 4) Multicomponent (*n* = 9)	PA behaviours	*Overall effect* Significant small effect (k=35^b^, g = 0.350, 95% (0.12, 0.58), *p* < 0.001) Heterogeneity: (Q_T_=1436.90, τ^2^ = 0.421, I^2^ = 98%) *Educational* No significant effect (k = 21, g = 0.225, 95% CI (-0.060, 0.509), *p* > 0.01) Heterogeneity: (τ^2^ = 0.105, I^2^ = 89.21) *Environmental* No significant effect (k = 4, g = 0.372, 95% CI (-0.301, 1.046), *p* > 0.01) Heterogeneity: (τ^2^ = 0.130, I^2^ = 71.60) *Multicomponent* Significant small to moderate effect (k = 9, g = 0.618, 95% CI (0.197, 1.039), *p* < 0.01) Heterogeneity: (τ^2^ = 0.827, I^2^ = 99.18)	NR
Voskuil et al. 2017 [61] *n* = 15 (5 reported on PA outcomes and are extracted)	Adolescent girls 8–12	Multicomponent (*n* = 5)	PA outcomes	Mixed (positive effect (*n* = 1/5))	NR
Adult women					
Amiri Farahani et al. 2015 [62] *n* = 9	Women 18–65	Community-based multicomponent (*n* = 9)	PA participation	Mixed (Positive effect (*n* = 7/9) (statistical significance *n* = 4))	NR
Madden et al. 2020 [31] *n* = 20 studies across 23 reports	Working women 33.2 ± 7.8 and 48.77 + 9.27	Workplace interventions Exercise (*n* = 5) Interrupted sitting (*n* = 1) Multicomponent (*n* = 14)	PA outcomes (Mean steps/day, Moderate steps/day, Weekly Leisure Activity Score, MVPA accelerometer, Total accelerometer counts, Workday sit time, Workday average sit time (hours), Workday sit-to-stand transitions, MET mins/week, VO_2_ peak, VO_2_ max)	Mixed (at least one positive effect (*n* = 12/17))	NR
Reed et al. 2017^m^ [30] *n* = 24	Working-Age Women 17–51 (83%)	Workplace interventions Single intervention strategy (*n* = 3) Multi intervention strategy (*n* = 21)	Minutes per week of MVPA METs per week *MET min/week*	*Minutes per week of MVPA* (*n* = 12) No significant effect (SMD = 0.38; 95% CI, (− 0.15, 0.92), *p* = 0.16) Heterogeneity: (I^2^ = 97%, *p* < 0.00001) *METs per week* (*n* = 3) No significant effect (SMD = 0.11; 95% CI (− 0.48, 0.71), *p* = 0.71) Heterogeneity: (I^2^ = 86%, *p* < 0.00001) *MET min/week* (*n* = 4) Significant effect (SMD = 2.07, 95% CI (1.44, 2.69), *p* < 0.00001) Heterogeneity: (I^2^ = 97%, *p* < 0.00001)	Very low
Mixed (both young and adolescent girls and adult women)					
Matheson et al. 2023 [63] *n* = 31 (4 reported on Movement behaviour outcomes and are extracted)	Girls and women 0–17 >35	Body image or movement-based interventions Unimodal (*n* = 1) Multimodal (*n* = 3)	Movement behaviour	*Overall effect* No effect (k = 4, d + = 0.036, 95% CI (-0.088, 0.161), 95% PI (-0.237, 0.310), *p* > 0.001) Heterogeneity: (I^2^ = 0.0%)	NR
Mothers and daughters					
Barnes et al. 2018 [64] *n* = 14 studies across 16 reports	Mothers and daughters 8–19 (daughters) 32-45.2 (mothers mean age)	Community-based intervention for mothers and daughters that targeted physical activity, fitness, nutrition, or adiposity	PA outcomes	*Mothers*: Mixed (significant positive group-by-time effect (*n* = 3/7)) *Daughters*: Mixed (significant positive group-by-time effect (*n* = 1/8))	NR
Brennan et al. 2021 [65] *n* = 11 (14 intervention arms)	Mothers and daughters 7–17 (daughters) 28–50 (mothers)	Described as mother and daughter interventions (no further detail reported)	PA levels	*Mothers*: Mixed (positive effect within group (*n* = 8/11) (statistical significance *n* = 6)) *Daughters*: Mixed (positive effect within group (*n* = 10/13) (statistical significance *n* = 8))	NR

*CI *confidence interval, *GRADE* Grading of Recommendations, Assessment, Development, and Evaluations, *MET* Metabolic Equivalent of Task, *MVPA* Moderate- to vigorous-physical activity, *NICE* The National Institute for Health and Care Excellence, *NR* not reported, *PA* physical activity, *PE* physical education, *SMD* standardised mean difference, *k* number of effect sizes, *g* effect size (Hedges' g); d+ = sample weighted average effect size

^m^meta-analysis

The majority of studies within the included systematic reviews were conducted in the USA [29–31, 54–57, 59–62, 64, 65]. Other countries where studies within the systematic reviews were conducted included Australia [29, 30, 54, 56, 57, 59–65], UK [29, 30, 53, 54, 56–60, 63], Iran [31, 56, 59, 60, 62, 64], and several European countries [29, 31, 54, 59, 60, 63].

There were a wide variety of outcomes reported which included participation in team sport [53], participation in physical activity [54, 62], attendance at physical education within school [58], physical activity behaviours [56, 60], movement behaviour [63], changes in physical activity levels [56, 65] or outcomes [29, 30, 57, 59, 61, 64, 65]. These outcomes often referred to the same concept and were used interchangeably across the systematic reviews to describe a range of outcome measures and units of measurement focusing on physical activity. Most of the systematic reviews (*n* = 12) combined and reported the results of both subjective (self-report physical activity questionnaires, diaries, logs) and objective outcome measures (accelerometer, pedometer) [29–31, 54–57, 59, 60, 62–65]. Commonly used units of measurements were specific minutes per week of MVPA, mean daily MVPA, Metabolic Equivalent of Task (MET) minutes per week, METs per week, Mean steps/day, Moderate steps/day and weekly leisure activity score [30, 31, 61]. Further details on outcome measures and units of measurements used can be found in additional file 6. 

### Methodological quality

The details of the critical appraisal scores for each included systematic review can be found in Table 3.

**Table 3:** JBI critical appraisal scores for systematic reviews and research syntheses

Study	JBI Appraisal items											Score
	1	2	3	4	5	6	7	8	9	10	11	
Allison et al. 2017 [53]	Y	Y	N	Y	N	U	U	Y	n/a	Y	Y	6
Amiri Farahani et al. 2015 [62]	Y	Y	U	N	U	Y	Y	Y	n/a	Y	Y	7
Barnes et al. 2018 [64]	Y	Y	N	U	U	Y	Y	Y	n/a	Y	Y	7
Biddle et al. 2014 [54, 55]	Y	Y	U	U	Y	Y	U	Y	Y	N	Y	7
Brennan et al. 2021 [65]	Y	Y	N	U	Y	Y	Y	Y	n/a	Y	Y	8
Camacho-Minano et al. 2011 [56]	Y	Y	U	U	N	U	Y	Y	n/a	Y	Y	6
Houle et al. 2020 [57]	Y	Y	U	U	N	Y	Y	N	n/a	Y	Y	6
Kelly et al. 2024 [58]	Y	Y	N	Y	Y	U	U	Y	n/a	Y	Y	7
Madden et al. 2020 [31]	Y	Y	Y	U	Y	N	Y	Y	n/a	Y	Y	8
Matheson et al. 2023 [63]	Y	Y	N	Y	Y	Y	Y	U	Y	Y	Y	9
NICE 2008 [29]	Y	Y	Y	U	Y	Y	U	Y	n/a	Y	Y	8
Owen et al. 2017 [59]	Y	Y	N	U	Y	U	Y	Y	Y	Y	Y	8
Pearson et al. 2015 [60]	Y	Y	N	U	Y	Y	U	Y	Y	N	Y	7
Reed et al. 2017 [30]	Y	Y	Y	Y	Y	Y	Y	Y	Y	Y	Y	11
Voskuil et al. 2017 [61]	Y	Y	Y	U	Y	U	U	Y	n/a	Y	Y	7

**Key:***Y* Yes, *N* No, *U* Unclear, *n/a* not applicable

1. Is the review question clearly and explicitly stated?

2. Were the inclusion criteria appropriate for the review question?

3. Was the search strategy appropriate?

4. Were the sources and resources used to search for studies adequate?

5. Were the criteria for appraising studies appropriate?

6. Was critical appraisal conducted by two or more reviewers independently?

7. Were there methods to minimize errors in data extraction?

8. Were the methods used to combine studies appropriate?

9. Was the likelihood of publication bias assessed?

10. Were recommendations for policy and/or practice supported by the reported data?

11. Were the specific directives for new research appropriate?

There are 11 items on the JBI critical appraisal checklist for systematic reviews and only one systematic review fulfilled all 11 criteria [30]. Only four of the reviews were assessed as having conducted an adequate search strategy including the use of subject headings or MESH terms as part of the search [29–31, 61]. Additionally, while all systematic reviews searched more than two bibliographic databases, only three reviews included a search for grey literature in the subject area [30, 58, 63]. Five systematic reviews did not use appropriate criteria for appraising studies as they developed their own appraisal tools or used questions from a range of pre-existing appraisal checklists without validating or piloting the new tool [53, 56, 57, 62, 64]. It was not always reported if the critical appraisal was performed by two or more reviewers [53, 56, 58, 59, 61] or if measures were taken to minimise errors in data extraction [53, 54, 58, 60, 61]. It was felt that 13 out of the 15 reviews used appropriate methods to combine studies. With regards to the data analysis only five of the reviews reported a meta-analysis [30, 54, 59, 60, 63]. The five systematic reviews that performed a meta-analysis assessed publication bias. Three reported small or negligible evidence of publication bias [54, 60, 63], while two found high probability [30, 59].

Regarding the designs of included studies within the systematic reviews, one systematic review did not report the type of primary research study included [58]. One systematic review only included cross-sectional and quasi-experimental studies [53]. Two systematic reviews only contained randomised controlled trials [61, 63], while the rest of the systematic reviews included a mixture of randomised and non-randomised studies (quasi-experimental, controlled trials, pre-post designs, among others) [29–31, 54, 56, 57, 59, 60, 62–65]. Based on the original author’s assessment, quality of the included primary research studies within the systematic reviews varied, with over half of them rated as poor quality or having high or moderate risk of bias. Further detail of the quality of included primary studies is extracted in additional file 6.

### Overlap

**Fig. 2:**
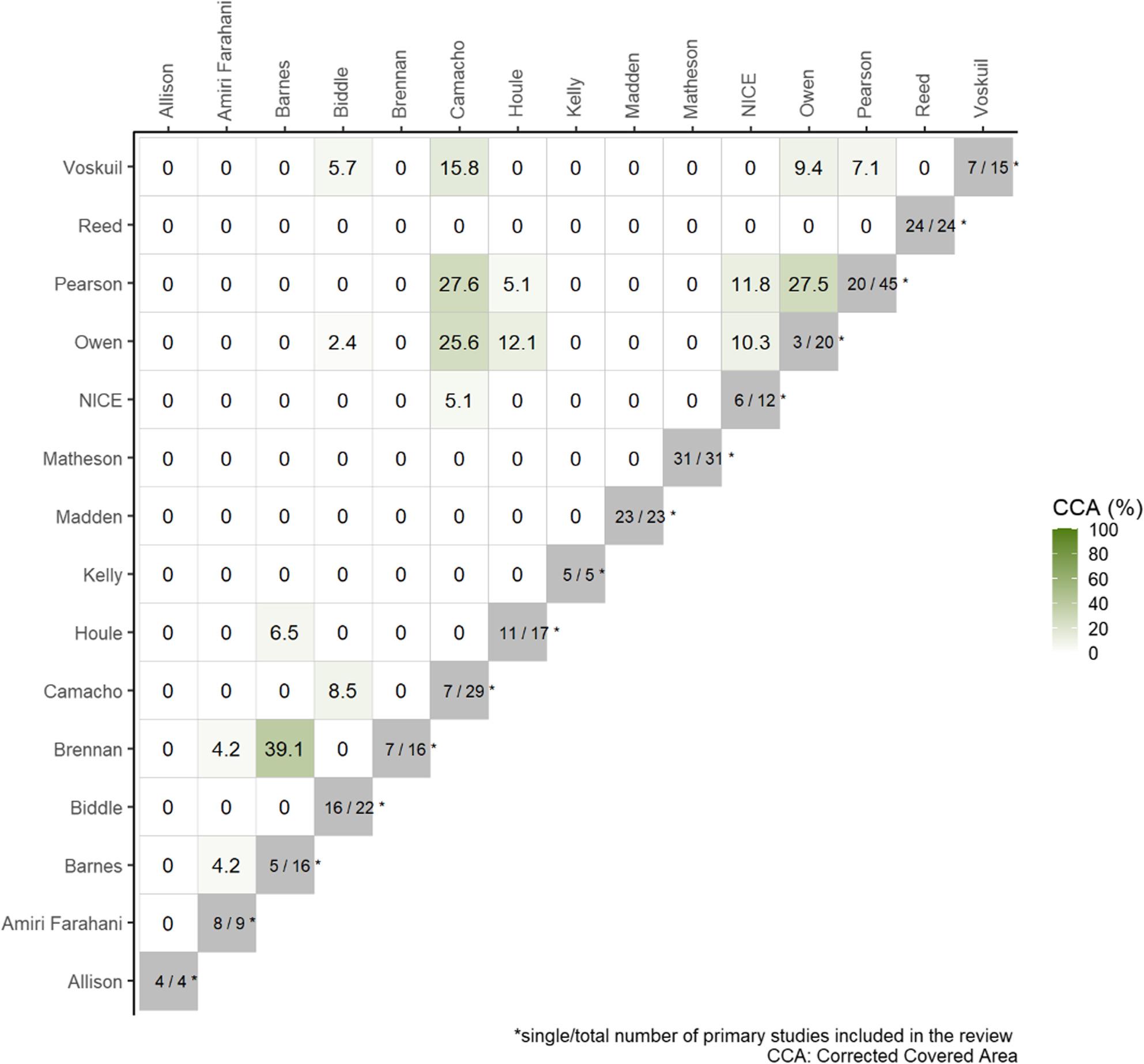
Heatmap of pairwise overlap between systematic reviews

The included systematic reviews contained 288 primary research reports, of which 222 were unique. Corrected covered area was 2.1%, indicating a slight overlap across all systematic reviews [66]. However, following pairwise comparison between systematic reviews, very high overlap (> 15%) was detected between five pairs, high (11–15%) between two pairs, moderate (6–10) between six pairs, and slight (0–5%) between six pairs. Overlap was detected between reviews focusing on similar populations, for example mothers and daughters, but none of the reviews completely overlapped. Pairwise overlap is depicted in Fig. 2.

#### Effectiveness of interventions for young and adolescent girls

Results across the nine systematic reviews which focused on young and adolescent girls found mixed effectiveness of interventions that support physical activity participation. None of the reviews focusing on young and adolescent girls described interventions focusing on managing physical activity participation throughout the menstrual cycle.

Six of the systematic reviews used narrative synthesis to summarise the results of primary research studies and three undertook meta-analysis. Those that used narrative synthesis found either mixed [29, 53, 56, 61] or no effect [57, 58]. No effect meant that all included primary studies failed to demonstrate measurable change in physical activity participation either at post-intervention or at follow-up. However, the systematic reviews showing no effect contained a very small number of primary studies, ranging from one to four. Mixed effect meant that some of the primary studies included in the systematic reviews showed a positive change in physical activity participation, while others failed to show an effect.

Regarding intervention types, the systematic review conducted by Houle et al. [57] that showed no effect focused on extra-curricular physical activity programmes, including physical activity only (dance) or multicomponent interventions (group discussions, motivational elements, text messaging, newsletters). The other review by Kelly et al. [58] that found no effect focused specifically on sporting role model interventions and identified one peer-reviewed publication [58]. Kelly et al. [58] additionally identified 15 interventions from the grey literature, although most had unavailable or unpublished programme evaluations, so effectiveness could not be inferred [58].

Out of the four systematic reviews with narrative synthesis reporting mixed results, one focused on team sport participation and the interventions included partnership working, grant funding, and multicomponent approaches (containing a mixture of staff training, action planning, grant funding, partnership working, resource provision, how-to guide, gender-specific research insights, and merchandise) [53]. Two reviews categorised interventions as single- (including educational) and/or multicomponent interventions, although it was unclear what the authors understood by single- and multicomponent [56, 61]. Interventions in these two systematic reviews often included a wide variety of components, such as various exercises, behavioural counselling, family involvement, health education, written material, self-monitoring, and environmental and policy change among others. The fourth systematic review categorised interventions as counselling, mediated (delivered via a medium such as computer, phone or printed materials), educational, or a combination of these [29]. All three intervention types or their combinations showed mixed effect in this systematic review, with the majority of mediated and counselling interventions demonstrating no effect [29].

The three systematic reviews that conducted meta-analysis found a significant small overall effect, although statistical heterogeneity was high, potentially due to the combination of different intervention types and delivery [54, 55, 59, 60]. Additionally, Owen et al. [59] identified an outlier study, and following its removal no significant effect was detected and heterogeneity substantially reduced. All three reviews conducted sub-group analyses based on intervention types, with Biddle et al. [54, 55] and Pearson et al. [60] categorising studies as educational, environmental and multicomponent (combination of educational and environmental). However, it was unclear how educational and environmental interventions were defined, and these also contained a wide selection of different elements, such as specialist led physical education, after-school or curriculum programme, behaviour modification lessons, and skill building, among others, adding to further clinical and statistical heterogeneity. Subgroup analyses in Owen et al. [59] were conducted based on two categories, single and multicomponent interventions. Similarly to the other two systematic reviews, these categories were not clearly defined, and multicomponent interventions contained a wide selection of elements, such as environmental adaptations, modified physical education lessons, educational and counselling sessions, extra-curricular physical activity, among others.

Results of the subgroup analyses shows that all three reviews found significant small effects for multicomponent interventions [54, 55, 59, 60], although two reviews still had significantly high heterogeneity [54, 55, 60]. Biddle et al. [54, 55] focused specifically on pre-adolescent girls, and reported a significant small effect for educational interventions, although statistical heterogeneity was high in this subgroup analysis. No significant effect was detected for single component interventions [59], and educational and environmental interventions [60] in adolescent girls.

Other characteristics reported in the nine systematic reviews focusing on young and adolescent girls included whether a theoretical approach, such as behaviour change or learning theories, was considered in the development of the interventions. In three reviews, theoretical approach was either not reported or not considered by the authors of the original primary studies [53, 57, 58]. The other six systematic reviews identified a wide variety of theories and models underpinning some of the interventions, and the most commonly used ones were social cognitive theory, social ecological model, and the transtheoretical model [29, 54–56, 59–61]. Theories used are detailed in additional file 7. The three systematic reviews with meta-analysis also conducted subgroup analysis based on whether a theory was used for intervention development [54, 59, 60]. Results from two of these systematic reviews found that interventions for adolescent girls with a theoretical approach were more effective than atheoretical ones [59, 60]. However, the systematic review focusing on pre-adolescents found that atheoretical interventions were more effective than those with a theoretical approach [54].

### Effectiveness of interventions for adult women

Three systematic reviews focused on interventions to support the physical activity participation of adult women [30, 31, 62], and none of these reported interventions on managing physical activity participation throughout the menstrual cycle. Two of these combined studies using narrative synthesis and one used a meta-analysis. The two systematic reviews reporting their findings using narrative synthesis found mixed effectiveness [31, 62]. These two systematic reviews included community-based multicomponent interventions [62], and workplace programmes categorised as exercise, interrupted sitting, and multicomponent interventions [31]. Community-based multicomponent interventions comprised of varied elements, such as social support, goal setting, self-monitoring, cultural facilitators, problem-solving training, media messages and economic incentives [62]. Multicomponent workplace programmes included elements, such as education, peer support, incentives, counselling, cognitive restructuring, problem solving and overcoming barriers, among others [31]. Madden et al. [31] did not provide a breakdown of whether exercise, interrupted sitting, or multicomponent interventions were more effective, although the authors did report that interventions that had technology as their main component, such as Nintendo Wii™ or treadmill, showed no effect on increasing physical activity outcomes. Both community-based multicomponent interventions and workplace programmes reported using behaviour change theories and models for their development with the most often reported being social cognitive theory and the social ecological model [31, 62]. Theories used are detailed in additional file 7.

The systematic review with meta-analysis of workplace interventions also found mixed results [30]. Studies included in the systematic review used different units of measurements for physical activity, specifically minutes per week of MVPA, METs per week, and MET min/week. Separate meta-analysis was conducted for each measurement unit and no significant effect for workplace interventions was detected in minutes per week of MVPA or METs per week. Statistically significant increase in physical activity was observed in MET min/week, although only a small number of studies (*n* = 4) reported this unit of measurement. Statistical heterogeneity was high in all three meta-analyses. Reed et al. [30] categorised workplace programmes as single and multi-intervention strategies, although no subgroup analysis was conducted based on whether an intervention was single or multicomponent. Additionally, all three meta-analyses contained a mixture of single and multicomponent interventions. Intervention elements reported were counselling, messages and emails for feedback, personal partner, team-goal setting, self-monitoring, educational sessions and reduction of perceived barriers, among others. However, Reed et al. [30] did not provide any details on whether the interventions were underpinned by any behaviour change or learning theories.

### Effectiveness of interventions for mixed population (young and adolescent girls and women)

One systematic review with a meta-analysis reported no statistically significant effect on movement behaviour following body image or movement-based interventions for young and adolescent girls or women [63]. Statistical heterogeneity was low, but the meta-analysis results were based on a small number of studies (*n* = 4). The interventions could further be categorised as unimodal and multimodal. Hatha yoga was provided as part of a unimodal intervention, while multimodal interventions focused on physical activity, strength-based approaches, or healthy body image [63]. The development of all four interventions was underpinned by a formal theory or framework, namely self-determination theory, exercise and self-esteem model, social cognitive theory and embodiment theory. The systematic review did not mention interventions focusing on managing physical activity participation throughout the menstrual cycle, and it was unclear whether the included studies contained both male and female participants, or females only.

### Effectiveness of mother and daughter interventions

Results from two systematic reviews focusing on mother and daughter interventions indicate mixed effectiveness [64, 65]. Both systematic review synthesised the evidence narratively, and neither of them conducted a meta-analysis. Positive effects of the interventions were mainly within group (pre- and post-intervention), with only a few studies within the systematic reviews showing significant between-group (group-by-time) effect [64, 65]. The investigated interventions were varied and included physical activity components, such as dance, fitness, interactive games, group walking, and material components, for example newsletters, booklets, certificates, DVDs, stickers, jump ropes, balls, weights, pedometers, and logbooks. Regarding a theoretical approach underpinning the interventions, Barnes et al. [64] did not report any formal theories, while Brennan et al. [65] mentioned social cognitive theory and the behaviour change wheel. Barnes et al. [64] concluded that multicomponent interventions and those that allowed mothers and daughters to participate together may lead to better physical activity participation. Brennan et al. [65] focused on behaviour change techniques and found that goal setting and information on health consequences were more promising to improve physical activity participation compared to others included in the systematic review. Regarding intervention components focusing on managing physical activity participation throughout the menstrual cycle, neither of the reviews mentioned or described such components.

### Certainty in the evidence

Only one systematic review focusing on adult women receiving workplace interventions conducted GRADE assessment, and the overall certainty in the evidence base was found very low [30]. Due to the majority of publications reporting findings narratively and the reviews with meta-analysis not reporting detailed risk of bias assessment, it was not possible to determine GRADE for the rest of the findings.

## Discussion

The aim of this rapid overview of reviews was to summarise systematic reviews investigating the effectiveness of interventions that could support women, girls, and people who menstruate to participate in physical activity, and most of the included systematic reviews indicate an overall mixed effect regardless of the target population. While these mixed results are mainly based on narrative reviews, where statistical pooling was not possible, the findings from systematic reviews with meta-analysis also indicate mixed effect. While significant small effects were detected for interventions designed for pre-adolescents and adolescents [54, 55, 60], in one review no statistically significant positive effect was detected after the removal of an outlier [59]. Subgroup analysis also indicates that single component interventions, educational, and environmental interventions may not increase physical activity participation significantly.

These results are similar to those observed in the wider literature not specifically focused on adolescent girls. Multiple systematic reviews exist that suggest that school-based interventions, such as educational interventions, do not increase physical activity participation significantly in the wider population including adolescent boys [67–69]. Additionally, there is no difference between the results of adolescent girls and boys [68, 69]. However, Schulze et al. [69] concluded that there was a need for sex/gender to be considered when developing or choosing intervention content and materials and measurement instruments. This is particularly important as this rapid overview of reviews also found that none of the interventions described in the included systematic reviews mentioned managing physical activity participation throughout the menstrual cycle, which should be a specific sex/gender consideration.

Recent reports suggest that young and adolescent girls’ interest in sports and physical activity reduces with menarche [10, 12, 20]. This is partly due to barriers related to menstruation, such as physical symptoms, low mood, fear and anxiety related to leaking, societal taboo, and beliefs related to physical activity during periods which often resulted in avoiding participation [21]. One recent UK survey reported that seven in 10 young and adolescent girls avoid being physically active during their periods [12]. This change in physical activity habits often lasts into adulthood, with some studies reporting as high a percentage as 66% of surveyed women avoiding physical activity during their periods [19]. Avoiding physical activity is also present among menstruating athletes of different levels (amateur, low or high level athletes) [22, 23]. Stopping training during their periods has been reported between 10% and 47.3% of female athletes depending on their sport level [22, 23]. This highlights the need for improving knowledge and resources around physical activity and the menstrual cycle. However, there is a lack of evidence-based guidelines worldwide regarding continuing to be physically active during the menstrual cycle [21], and the findings of this rapid overview of reviews support this. The lack of mention regarding the menstrual cycle in 15 systematic reviews specifically focusing on girls and women, indicates a gap in the physical activity promotion literature. This lack of evidence is significant, as insufficient physical activity is a risk factor for non-communicable diseases [1], whilst evidence suggests that exercise may be an effective strategy for managing premenstrual syndrome [70], and dysmenorrhea [71].

A recent report has also identified a lack of evidence on interventions focusing on managing physical activity throughout the menstrual cycle with only two primary research studies and an organisational report identified [32]. These studies and reports focused on interventions, such as social-media-based support for premenstrual syndrome and physical activity [72], codesigning menstrual technologies with adolescents [73], and the provision of free period products in deprived areas as part of a multicomponent programme called Big Sister [74]. While some of these studies were more exploratory, social-media-based support was found to increase physical activity [72], and the Big Sister project reported that 44% of adolescent girls engaged in the programme were more physically active [74]. These findings are promising, although small sample size and lack of robust evaluation methods used highlight the need for more research. Future research should focus on developing and evaluating interventions that address barriers related to the menstrual cycle. Additionally, while the above interventions focus on helping individuals to become more physically active, further initiatives may also be needed to reduce societal taboo around the menstrual cycle, which is often reported as a barrier [21]. Policymakers will need to consider guidance that could help reduce societal taboo around the menstrual cycle and menstruation.

While menstruation and the menstrual cycle can have a significant impact on young and adolescent girls’ physical activity participation, other factors can also act as a barrier. There is a significant evidence-base suggesting that lack of family and peer support, time limitations, gender bias, body image, and perceived competence can also act as barriers [13, 14]. Therefore, approaches that target multiple inhibiting factors may be necessary. Recommendations that have been made in the literature include, addressing gender norms in the curriculum, training for teachers and professionals, and environmental changes [14]. While environmental and single component approaches did not seem to be effective individually [54, 55, 59, 60], as supported by the wider evidence-base [75], multicomponent interventions were found more effective across the reviews. These multicomponent interventions combined educational and environmental elements which often included skill building, behaviour modification lessons, and modified physical education that could be tailored for the individual or a group of young and adolescent girls [59, 60]. In some cases, providing choice and more opportunities for physical activity was also implemented in the forms of after-school programmes [59, 60]. This supports that multiple factors may need to be targeted for young and adolescent girls to be physically active.

Regarding adult women, similar mixed effectiveness of interventions aiming to improve physical activity participation was identified as for young and adolescent girls. Multicomponent and individualised interventions seemed to be more effective than unimodal interventions lacking tailoring [31]. A multicomponent approach is crucial as adult women face diverse barriers to physical activity, some of which may be similar to those experienced by young and adolescent girls’, such as gender bias, body image, lack of family and peer support, and time [15, 43]. However, other barriers may be specific to adults, such as family and domestic duties [15, 43]. Additionally, adult women also experience menstruation and the menstrual cycle as barriers, which is not considered in any of the included systematic reviews in this rapid overview of reviews [19]. Tailoring interventions by creating personalised physical activity plans or providing education and support with problem solving and overcoming barriers specific to the individual may help women become more active [31]. Future research should focus on multicomponent interventions tailored to the needs of adult women, particularly considering the menstrual cycle.

Regarding quality of the evidence, most included systematic reviews combined a wide variety of outcome measures and units of measurements as part of their synthesis. This could have an impact on the findings as results of objective and subjective measures capturing different dimensions of physical activity (frequency, duration, or intensity) were often used to provide overall conclusions in the systematic reviews and could have led to the mixed effectiveness found. The systematic review with meta-analysis focusing on adult women in the workplace found different results with different units of measurement used (Minutes per week MVPA, MET min/week) [30]. As separate analysis containing different interventions was conducted for each unit of measurement, it is possible that the differing results were simply reflecting the effectiveness of the interventions involved. However, research from the wider evidence base suggests that using various analytical methods and units of measurement may detect change in physical activity participation differently [48, 76, 77]. Hence, there is a need for more research using uniform and reliable data collection and analytical methods that are sensitive to detect changes in specific populations, inclusive of women, girls, and people who menstruate.

Explanation for mixed effect across the 15 systematic reviews may relate to interventions and components often not being defined. Thus, it is important to note that it is challenging to make conclusions due to the lack of definitions provided. Additionally, diverse and undefined interventions were frequently being grouped based on the delivery setting, for example school or community. This potentially led to the high clinical and statistical heterogeneity and difficulty in combining data in a meta-analysis. Although not specifically investigated for this rapid overview of reviews, primary studies across the reviews also differed in delivery method, theoretical approach, profession of instructors, duration, and intensity, leading to further heterogeneity across the studies. Moreover, review authors reported high or moderate risk of bias in the majority of included studies, which could lead to varying results and a lack of certainty in the evidence. Only one systematic review conducted GRADE assessment, which found very low certainty in the evidence for workplace interventions for adult women [30]. Future research will need to develop robust study designs and define and describe interventions for replicability and generalisability.

### Strength and limitations of the review

While established rapid review guidance was followed for this review, it still has some limitations. Even though a comprehensive search strategy was developed across multiple databases, it is possible that relevant reviews may have been missed due to focusing our text word searches on terms related to girls, women, female or assigned female at birth, menstruation, and people who menstruate. This means systematic reviews that did not mention these or similar terms in the title and abstract may not have been identified via database searches. However, forward and backward citation searching were completed and a thorough grey literature search across multiple organisational and government website was conducted, ensuring that as many relevant systematic reviews were identified as possible.

The advantage of overviews of reviews is that they provide a breadth of evidence that would not be possible to cover in a single systematic or rapid review. However, a potential limitation of this approach is that review of the individual primary research studies is not conducted. This means that it is possible that details of interventions and the populations may have been missed. However, included studies tables within the systematic reviews and additional materials were thoroughly checked, and titles of primary research studies were screened to ensure that no significant information was missed. An additional limitation may be that while there was only a slight overlap across all 15 systematic reviews, very high pairwise overlap existed between reviews focusing on mother and daughter interventions, and some of the publications focusing on adolescent girls. This could potentially cause some overestimation in the breadth of the evidence.

Another possible limitation of this rapid overview of reviews is that it did not include girls prior to menarche, pregnant, postpartum, menopausal and post-menopausal women. Hence, it is possible that other relevant interventions, that may also be useful for the population in this rapid overview of reviews and could influence the findings, have been missed. This review also did not include systematic reviews that specifically targeted those living with chronic conditions, obesity, poor nutrition or with a smoking habit. These populations may require more specialist support potentially by healthcare services, thus the findings in this rapid overview of reviews may not be generalisable to them.

Only two systematic reviews reported including ethnic minorities, and none of the publications mentioned people who menstruate but do not identify as a girl or a woman. As these populations often face specific barriers, such as cultural differences or stigma, their needs and interventions that could support them to participate in physical activity should be investigated. Furthermore, while no geographical limits were applied in this rapid overview of reviews, most evidence originated from high income countries, such as the USA, Australia, and the UK. Thus, the findings may not be generalisable to low- and middle-income countries where certain barriers impacting on women, girls and people who menstruate may differ due to different social and cultural expectations.

## Conclusions

A substantial systematic review evidence base focusing on interventions aiming to support physical activity participation for women and girls exists, although findings are mixed and the certainty in the evidence is often very low. Additionally, these women and girls specific systematic reviews do not consider interventions which are sex/gender specific including management of the menstrual cycle during physical activity. High quality research is needed to identify interventions that could help girls, women, and people who menstruate be physically active, with consideration to barriers related to the menstrual cycle. Additionally, policymakers will need to consider guidance that could help ease the societal taboo around menstruation.

## Supplementary Information

Additional file 1: PRISMA checklist Preferred Reporting Items for Systematic reviews and Meta-Analyses (PRISMA) 2020 checklist.

Additional file 2: PRIOR checklist Reporting Items for Overviews of Reviews (PRIOR).

Additional file 3: Search strategies Comprehensive search strategies across all the included databases.

Additional file 4: Websites of key third sector and government organisations A table detailing the websites of key third sector and government organisations.

Additional file 5: List of excluded reports Table of reports excluded on full text screening with the reason for exclusion.

Additional file 6: Data extraction Data extraction table containing the characteristics and findings of included systematic reviews.

Additional file 7: Table of interventions and behaviour change theories A table containing components and underpinning behaviour change theories of interventions included in the systematic reviews.

## Data Availability

All data analysed during this rapid overview of reviews are included in this published article and its supplementary information files. The search strategy is included within the additional files, but has also been shared on searchRxiv. The links to the search strategy can be found below: MEDLINE https://doi.org/10.1079/searchRxiv.2024.00673 AMED https://doi.org/10.1079/searchRxiv.2024.00674 CINAHL https://doi.org/10.1079/searchRxiv.2024.00675 Cochrane https://www.cabidigitallibrary.org/doi/10.1079/searchRxiv.2024.00676 Emcare https://doi.org/10.1079/searchRxiv.2024.00677 Epistemonikos https://doi.org/10.1079/searchRxiv.2024.00678 SportDiscus https://doi.org/10.1079/searchRxiv.2024.00679.

## Abbreviations

GRADE: Grading of Recommendations, Assessment, Development and Evaluation
OSF: Open Science Framework
PICO: Population, Intervention, Comparator, Outcome
PRISMA: Preferred Reporting Items for Systematic reviews and Meta-Analyses
PRIOR: Reporting Items for Overviews of Reviews
MVPA: Moderate to vigorous physical activity
MET: Metabolic Equivalent of Task
NICE: The National Institute for Health and Care Excellence
PA: Physical activity
PE: Physical education
UK: United Kingdom
USA: United States of America
WHO: World Health Organisation
