# Supplementary material for: Interventions that support women, girls, and people who menstruate to participate in physical activity: a rapid overview of reviews
Source: BMC Public Health. 2026 Mar 27;26:1472. doi: 10.1186/s12889-026-27122-9 (PMC13147802; doi:10.1186/s12889-026-27122-9)
Supplement: Supplementary file 3 — Additional file 3: Search strategies Comprehensive search strategies across all the included databases. [file 12889_2026_27122_MOESM3_ESM.docx]

**Additional file 3: Search strategies**

**Ovid MEDLINE(R) ALL <1946 to March 27, 2024>**

| **#** | **Query** | **Results from 28 Mar 2024** |
| --- | --- | --- |
| 1 | (wom#n or female* or girl*).ti. | 484,575 |
| 2 | exp Menstruation Disturbances/ | 29,708 |
| 3 | exp Menstrual Cycle/ | 35,971 |
| 4 | (menstrua* or menses or dysmenorrhea or menorrhagia or menarche or eumenorrh*).tw. | 73,582 |
| 5 | (premenstrual adj2 (syndrome* or tension*)).tw. | 3,219 |
| 6 | 1 or 2 or 3 or 4 or 5 | 565,249 |
| 7 | exp Exercise/ | 254,843 |
| 8 | exp Sports/ | 221,903 |
| 9 | exp "Physical Education and Training"/ | 14,263 |
| 10 | (exercise* or sport*).tw. | 448,763 |
| 11 | (physical* adj2 (activ* or inactiv* or train* or education)).tw. | 183,919 |
| 12 | (aerobic* or walk* or swim* or jog* or run* or cycl* or bicyc* or bike or biking or dance or dancing or gymnastic*).tw. | 1,916,057 |
| 13 | (yoga or tai chi or qi gong or pilates).tw. | 9,817 |
| 14 | (tennis or golf* or football or soccer or rugby or netball or hockey or cricket or basketball or volleyball).tw. | 45,146 |
| 15 | 7 or 8 or 9 or 10 or 11 or 12 or 13 or 14 | 2,480,473 |
| 16 | (participat* or engage* or uptake* or attend* or promot*).tw. | 2,801,558 |
| 17 | (involv* or encourag* or increas* or adher* or maintain* or sustain*).ti. | 668,960 |
| 18 | (behavio?r adj3 change*).tw. | 47,433 |
| 19 | ((decreas* or reduc* or discourag*) adj2 (sedentary or inactiv*)).tw. | 5,415 |
| 20 | 16 or 17 or 18 or 19 | 3,406,458 |
| 21 | exp "Systematic Review"/ | 256,301 |
| 22 | exp Meta-Analysis/ | 197,701 |
| 23 | exp Systematic Reviews as Topic/ | 12,944 |
| 24 | exp Meta-Analysis as Topic/ | 29,370 |
| 25 | (systematic review* or meta-analysis or review*).pt. | 3,480,086 |
| 26 | (systematic adj3 (review* or overview*)).ti,ab,kf. | 342,509 |
| 27 | (quantitative adj3 (review* or overview* or synthes*)).ti,ab,kf. | 9,085 |
| 28 | (meta-analy* or metaanaly* or meta-synthes#s or metasynthes#s).tw. | 301,458 |
| 29 | 21 or 22 or 23 or 24 or 25 or 26 or 27 or 28 | 3,585,935 |
| 30 | 6 and 15 and 20 and 29 | 1,058 |
| **31** | limit 30 to (english language and yr="2008 -Current") | **661** |

**Ovid Emcare <1995 to 2024 Week 12>**

| **#** | **Query** | **Results from 28 Mar 2024** |
| --- | --- | --- |
| 1 | (wom#n or female* or girl*).ti. | 204,166 |
| 2 | exp menstrual cycle/ | 15,740 |
| 3 | exp menstruation disorder/ | 13,823 |
| 4 | (menstrua* or menses or dysmenorrhea or menorrhagia or menarche or eumenorrh*).tw. | 21,889 |
| 5 | (premenstrual adj2 (syndrome* or tension*)).tw. | 1,087 |
| 6 | 1 or 2 or 3 or 4 or 5 | 232,551 |
| 7 | exp exercise/ | 144,103 |
| 8 | exp sport/ | 94,112 |
| 9 | exp physical education/ | 7,102 |
| 10 | exp physical activity/ | 193,657 |
| 11 | (exercise* or sport*).tw. | 237,073 |
| 12 | (physical* adj2 (activ* or inactiv* or train* or education)).tw. | 116,087 |
| 13 | (aerobic* or walk* or swim* or jog* or run* or cycl* or bicyc* or bike or biking or dance or dancing or gymnastic*).tw. | 368,612 |
| 14 | (yoga or tai chi or qi gong or pilates).tw. | 7,247 |
| 15 | (tennis or golf* or football or soccer or rugby or netball or hockey or cricket or basketball or volleyball).tw. | 33,421 |
| 16 | 7 or 8 or 9 or 10 or 11 or 12 or 13 or 14 or 15 | 739,454 |
| 17 | (participat* or engage* or uptake* or attend* or promot*).tw. | 940,748 |
| 18 | (involv* or encourag* or increas* or adher* or maintain* or sustain*).ti. | 170,681 |
| 19 | (behavio?r adj3 change*).tw. | 24,548 |
| 20 | ((decreas* or reduc* or discourag*) adj2 (sedentary or inactiv*)).tw. | 1,982 |
| 21 | *behavior change/ | 5,529 |
| 22 | 17 or 18 or 19 or 20 or 21 | 1,095,825 |
| 23 | exp "systematic review"/ | 127,862 |
| 24 | exp meta analysis/ | 61,388 |
| 25 | exp "systematic review (topic)"/ | 8,710 |
| 26 | exp "meta analysis (topic)"/ | 10,552 |
| 27 | (systematic review* or meta-analysis or review*).pt. | 877,445 |
| 28 | (systematic adj3 (review* or overview*)).ti,ab,kf. | 174,731 |
| 29 | (quantitative adj3 (review* or overview* or synthes*)).ti,ab,kf. | 4,668 |
| 30 | (meta-analy* or metaanaly* or meta-synthes#s or metasynthes#s).tw. | 134,589 |
| 31 | 23 or 24 or 25 or 26 or 27 or 28 or 29 or 30 | 1,015,765 |
| 32 | 6 and 16 and 22 and 31 | 636 |
| 33 | limit 32 to (english language and yr="2008 -Current") | **409** |

**AMED (Allied and Complementary Medicine) <1985 to October 2023>**

| **#** | **Query** | **Results from 28 Mar 2024** |
| --- | --- | --- |
| 1 | (wom#n or female* or girl*).ti. | 6,378 |
| 2 | exp Menstrual cycle/ | 157 |
| 3 | exp Menstruation disorders/ | 663 |
| 4 | (menstrua* or menses or dysmenorrhea or menorrhagia or menarche or eumenorrh*).tw. | 1,019 |
| 5 | (premenstrual adj2 (syndrome* or tension*)).tw. | 193 |
| 6 | 1 or 2 or 3 or 4 or 5 | 7,402 |
| 7 | exp Exercise/ | 10,727 |
| 8 | exp Sports/ | 6,802 |
| 9 | exp Physical education/ | 2,183 |
| 10 | (exercise* or sport*).tw. | 37,478 |
| 11 | (physical* adj2 (activ* or inactiv* or train* or education)).tw. | 8,168 |
| 12 | (aerobic* or walk* or swim* or jog* or run* or cycl* or bicyc* or bike or biking or dance or dancing or gymnastic*).tw. | 28,464 |
| 13 | (yoga or tai chi or qi gong or pilates).tw. | 2,081 |
| 14 | (tennis or golf* or football or soccer or rugby or netball or hockey or cricket or basketball or volleyball).tw. | 3,224 |
| 15 | 7 or 8 or 9 or 10 or 11 or 12 or 13 or 14 | 62,020 |
| 16 | (participat* or engage* or uptake* or attend* or promot*).tw. | 35,334 |
| 17 | (involv* or encourag* or increas* or adher* or maintain* or sustain*).ti. | 4,966 |
| 18 | (behavio?r adj3 change*).tw. | 725 |
| 19 | ((decreas* or reduc* or discourag*) adj2 (sedentary or inactiv*)).tw. | 99 |
| 20 | 16 or 17 or 18 or 19 | 39,887 |
| 21 | exp Meta analysis/ | 427 |
| 22 | (systematic review* or meta-analysis or review*).pt. | 14,041 |
| 23 | (systematic adj3 (review* or overview*)).ti,ab. | 7,324 |
| 24 | (quantitative adj3 (review* or overview* or synthes*)).ti,ab. | 210 |
| 25 | (meta-analy* or metaanaly* or meta-synthes#s or metasynthes#s).tw. | 3,925 |
| 26 | 21 or 22 or 23 or 24 or 25 | 16,805 |
| 27 | 6 and 15 and 20 and 26 | 12 |
| 28 | limit 27 to (english and yr="2008 -Current") | **10** |

**CINAHL (Cumulative Index to Allied Health Nursing) EBSCO Host**

| **#** | **Query** | **Results from 28 Mar 2024** |
| --- | --- | --- |
| S1 | TI (wom?n or female* or girl*) | 204,994 |
| S2 | (MH “Menstruation Disorders+”) | 8909 |
| S3 | (MH "Menstrual Cycle+") | 8065 |
| S4 | TI ( menstrua* or menses or dysmenorrhea or menorrhagia or menarche or eumenorrh* ) OR AB ( menstrua* or menses or dysmenorrhea or menorrhagia or menarche or eumenorrh* ) | 16,777 |
| S5 | TI ( premenstrual N2 (syndrome* or tension*) ) OR AB ( premenstrual N2 (syndrome* or tension*) ) | 1002 |
| S6 | 1 or 2 or 3 or 4 or 5 | 223,097 |
| S7 | (MH "Exercise+") | 132,067 |
| S8 | (MH "Sports+") | 92,084 |
| S9 | (MH "Physical Activity") | 53,455 |
| S10 | (MH "Physical Education and Training+") | 4,842 |
| S11 | TI ( exercise* or sport* ) OR AB ( exercise* or sport* ) | 188,810 |
| S12 | TI ( physical* N2 (activ* or inactiv* or train* or education) ) OR AB ( physical* N2 (activ* or inactiv* or train* or education) ) | 92,610 |
| S13 | TI ( aerobic* or walk* or swim* or jog* or run* or cycl* or bicyc* or bike or biking or dance or dancing or gymnastic* ) OR AB ( aerobic* or walk* or swim* or jog* or run* or cycl* or bicyc* or bike or biking or dance or dancing or gymnastic* ) | 231,168 |
| S14 | TI ( yoga or "tai chi" or "qi gong" or pilates ) OR AB ( yoga or "tai chi" or "qi gong" or pilates ) | 8,460 |
| S15 | TI ( tennis or golf* or football or soccer or rugby or netball or hockey or cricket or basketball or volleyball ) OR AB ( tennis or golf* or football or soccer or rugby or netball or hockey or cricket or basketball or volleyball ) | 24,440` |
| S16 | 7 or 8 or 9 or 10 or 11 or 12 or 13 or 14 or 15 | 544,072 |
| S17 | TI ( participat* or engage* or uptake* or attend* or promot* ) OR AB ( participat* or engage* or uptake* or attend* or promot* ) | 656,191 |
| S18 | TI (involv* or encourag* or increas* or adher* or maintain* or sustain*) | 164,644 |
| S19 | TI behavio#r N3 change* OR AB behavio#r N3 change* | 19,570 |
| S20 | TI ( decreas* or reduc* or discourag*) N2 (sedentary or inactiv* ) OR AB ( decreas* or reduc* or discourag*) N2 (sedentary or inactiv* ) | 1615 |
| S21 | (MM "Behavioral Changes") | 5498 |
| S22 | 17 or 18 or 19 or 20 or 21 | 813,203 |
| S23 | (MH "Systematic Review") | 133,237 |
| S24 | (MH "Meta Analysis") | 73,641 |
| S25 | PT ("systematic review*" or "meta-analysis" or review*) | 526,228 |
| S26 | TI ( systematic N3 (review* or overview* ) OR AB ( systematic N3 (review* or overview* ) | 161,427 |
| S27 | TI ( quantitative N3 (review* or overview* or synthes* ) OR AB ( quantitative N3 (review* or overview* or synthes* ) | 4030 |
| S28 | TI ( meta-analy* or metaanaly* or meta-synthes?s or metasynthes?s ) OR AB ( meta-analy* or metaanaly* or meta-synthes?s or metasynthes?s ) | 117,942 |
| S29 | 23 or 24 or 25 or 26 or 27 or 28 | 629,025 |
| S30 | 6 and 16 and 22 and 29 | 12 |
| S31 | limit 30 to publication date: 20080101 – 20240431; English Language | **257** |

**SportDiscus EBSCO Host 26/03/2024**

| **ID#** | **Search Terms** | **Results** |
| --- | --- | --- |
| S26 | S6 AND S15 AND S20 AND S25  **Limiters** - Published: 20080101-; Language: English | **109** |
| S25 | S21 OR S22 OR S23 OR S24 | 22,260 |
| S24 | TI "rapid review*" OR AB "rapid review*" OR KW "rapid review*" | 68 |
| S23 | TI ( "meta-analy*" or "metaanaly*" or "meta-synthes#s" or "metasynthes#s" ) OR AB ( "meta-analy*" or "metaanaly*" or "meta-synthes#s" or "metasynthes#s" ) OR KW ( "meta-analy*" or "metaanaly*" or "meta-synthes#s" or "metasynthes#s" ) | 11,680 |
| S22 | TI ( "quantitative" N3 ("review*" or "overview*" or "synthes*") ) OR AB ( "quantitative" N3 ("review*" or "overview*" or "synthes*") ) OR KW ( "quantitative" N3 ("review*" or "overview*" or "synthes*") ) | 467 |
| S21 | TI ( "systematic" N3 ("review*" or "overview*") ) OR AB ( "systematic" N3 ("review*" or "overview*") ) OR KW ( "systematic" N3 ("review*" or "overview*") ) | 17,694 |
| S20 | S16 OR S17 OR S18 OR S19 | 453,412 |
| S19 | TI ( (“decreas*” or “reduc*” or “discourag*”) N2 (“sedentary” or “inactiv*”) ) OR AB ( (“decreas*” or “reduc*” or “discourag*”) N2 (“sedentary” or “inactiv*”) ) OR KW ( (“decreas*” or “reduc*” or “discourag*”) N2 (“sedentary” or “inactiv*”) ) | 754 |
| S18 | TI "behavio#r" N3 "change*" | 855 |
| S17 | TI “involv*” or “encourag*” or “increas*” or “adher*” or “maintain*” or “sustain*” | 298,784 |
| S16 | TI ( "participat*" or "engage*" or "uptake*" or "attend*" or "promot*" ) OR AB ( "participat*" or "engage*" or "uptake*" or "attend*" or "promot*" ) OR KW ( "participat*" or "engage*" or "uptake*" or "attend*" or "promot*" ) | 214,249 |
| S15 | S7 OR S8 OR S9 OR S10 OR S11 OR S12 OR S13 OR S14 | 1,290,177 |
| S14 | TI ( tennis or golf* or football or "soccer" or "rugby" or "netball" or "hockey" or "cricket" or "basketball" or "volleyball" ) OR AB ( "soccer" or "rugby" or "netball" or "hockey" or "cricket" or "basketball" or "volleyball" ) OR KW ( "soccer" or "rugby" or "netball" or "hockey" or "cricket" or "basketball" or "volleyball" ) | 276,905) |
| S13 | TI ( "yoga" or "tai chi" or "qi gong" or "pilates" ) OR AB ( "yoga" or "tai chi" or "qi gong" or "pilates" ) OR KW ( "yoga" or "tai chi" or "qi gong" or "pilates" ) | 13,149 |
| S12 | TI ( "aerobic*" or "walk*" or "swim*" or "jog*" or "run*" or "cycl*" or "bicyc*" or "bike" or "biking" or "dance" or "dancing" or "gymnastic*" ) OR AB ( "aerobic*" or "walk*" or "swim*" or "jog*" or "run*" or "cycl*" or "bicyc*" or "bike" or "biking" or "dance" or "dancing" or "gymnastic*" ) OR KW ( "aerobic*" or "walk*" or "swim*" or "jog*" or "run*" or "cycl*" or "bicyc*" or "bike" or "biking" or "dance" or "dancing" or "gymnastic*" ) | 371,368 |
| S11 | TI ( "physical*" N2 ("activ*" or "inactiv*" or "train*" or "education") ) OR AB ( "physical*" N2 ("activ*" or "inactiv*" or "train*" or "education") ) OR KW ( "physical*" N2 ("activ*" or "inactiv*" or "train*" or "education") ) | 132,003 |
| S10 | TI ( "exercise*" or "sport*" ) OR AB ( "exercise*" or "sport*" ) OR KW ( "exercise*" or "sport*" ) | 644,260 |
| S9 | (DE "PHYSICAL education" OR DE "COACHING (Athletics)" OR DE "COLLEGE sports" OR DE "DRILLS (Practice)" OR DE "FIELD days (Education)" OR DE "FUNCTIONAL training" OR DE "MOTOR learning" OR DE "MOVEMENT education" OR DE "MUSIC in physical education" OR DE "PHYSICAL Education Attitude Inventory" OR DE "PHYSICAL Education Teacher Assessment Instrument" OR DE "PHYSICAL education (Elementary)" OR DE "PHYSICAL education (Middle school)" OR DE "PHYSICAL education (Primary)" OR DE "PHYSICAL education (Se [...](javascript:showHistoryTerm('ctl00_ctl00_FindField_FindField_historyControl_HistoryRepeater_ctl17_ellipsis',true)) | 170,648 |
| S8 | DE "SPORTS" OR DE "AERODYNAMICS in sports" OR DE "AERONAUTICAL sports" OR DE "AGE & sports" OR DE "AMATEUR sports" OR DE "ANIMAL sports" OR DE "ANTISEMITISM in sports" OR DE "AQUATIC sports" OR DE "BALL games" OR DE "BALLISTICS in sports" OR DE "BASEBALL" OR DE "BIOMECHANICS in sports" OR DE "COLLEGE sports" OR DE "COMBAT sports" OR DE "COMMUNICATION in sports" OR DE "CONTACT sports" OR DE "CROSS-training (Sports)" OR DE "DISC golf" OR DE "DISCRIMINATION in sports" OR DE "DOG sports" OR DE "DOPI [...](javascript:showHistoryTerm('ctl00_ctl00_FindField_FindField_historyControl_HistoryRepeater_ctl18_ellipsis',true)) | 288,641 |
| S7 | DE "EXERCISE" OR DE "ABDOMINAL exercises" OR DE "AEROBIC exercises" OR DE "ANAEROBIC exercises" OR DE "AQUATIC exercises" OR DE "ARM exercises" OR DE "BACK exercises" OR DE "BREATHING exercises" OR DE "BREEMA" OR DE "BUTTOCKS exercises" OR DE "CALISTHENICS" OR DE "CHAIR exercises" OR DE "CHEST exercises" OR DE "CIRCUIT training" OR DE "COMPOUND exercises" OR DE "COOLDOWN" OR DE "DO-in" OR DE "EXERCISE adherence" OR DE "EXERCISE for children" OR DE "EXERCISE for girls" OR DE "EXERCISE for men" OR [...](javascript:showHistoryTerm('ctl00_ctl00_FindField_FindField_historyControl_HistoryRepeater_ctl19_ellipsis',true)) | 190,990 |
| S6 | S1 OR S2 OR S3 OR S4 OR S5 | 87,047 |
| S5 | TI ( "premenstrual" N2 ("syndrome*" or "tension*") ) OR AB ( "premenstrual" N2 ("syndrome*" or "tension*") ) OR KW ( "premenstrual" N2 ("syndrome*" or "tension*") | 230 |
| S4 | TI ( "menstrua*" or "menses" or "dysmenorrhea" or "menorrhagia" or "menarche" or "eumenorrh*" ) OR AB ( "menstrua*" or "menses" or "dysmenorrhea" or "menorrhagia" or "menarche" or "eumenorrh*" ) OR KW ( "menstrua*" or "menses" or "dysmenorrhea" or "menorrhagia" or "menarche" or "eumenorrh*" ) | 4,355 |
| S3 | DE "MENSTRUATION" OR DE "MENSTRUATION disorders" OR DE "PREMENSTRUAL syndrome" OR DE "MENSTRUAL cycle" OR DE "MENARCHE" OR DE "HUMAN reproduction" | 3,376 |
| S2 | TI (“wom#n” or “female*” or “girl*”) | 52,577 |
| S1 | DE "WOMEN" OR DE "OVERWEIGHT women" OR DE "PREGNANT women" OR DE "SEDENTARY women" OR DE "WOMEN college students" OR DE "WOMEN dancers" OR DE "WOMEN with disabilities" | 48,338 |

**Cochrane: 26/03/2024**

| **ID** | **Search** | **Hits** |
| --- | --- | --- |
| #1 | (wom?n OR female* OR girl*):ti | 70837 |
| #2 | MeSH descriptor: [Menstruation Disturbances] explode all trees | 2657 |
| #3 | MeSH descriptor: [Menstrual Cycle] explode all trees | 2418 |
| #4 | (menstrua* or menses or dysmenorrhea or menorrhagia or menarche or eumenorrh*):ti,ab,kw | 17341 |
| #5 | (premenstrual NEAR/2 (syndrome* or tension*)):ti,ab,kw | 1291 |
| #6 | #1 OR #2 OR #3 OR #4 OR #5 | 84934 |
| #7 | MeSH descriptor: [Exercise] explode all trees | 38461 |
| #8 | MeSH descriptor: [Sports] explode all trees | 21975 |
| #9 | MeSH descriptor: [Physical Education and Training] explode all trees | 1907 |
| #10 | (exercise* or sport*):ti,ab,kw | 149607 |
| #11 | (physical* NEAR/2 (activ* or inactiv* or train* or education)):ti,ab,kw | 53642 |
| #12 | (aerobic* or walk* or swim* or jog* or run* or cycl* or bicyc* or bike or biking or dance or dancing or gymnastic*):ti,ab,kw | 192138 |
| #13 | (yoga or tai chi or qi gong or pilates):ti,ab,kw | 8398 |
| #14 | (tennis or golf* or football or soccer or rugby or netball or hockey or cricket or basketball or volleyball):ti,ab,kw | 6487 |
| #15 | #7 OR #8 OR #9 OR #10 OR #11 OR #12 OR #13 OR #14 | 314719 |
| #16 | (participat* or engage* or uptake* or attend* or promot*):ti,ab,kw | 276913 |
| #17 | (involv* or encourag* or increas* or adher* or maintain* or sustain*):ti | 47884 |
| #18 | (behavio?r NEAR/3 change*):ti,ab,kw | 11996 |
| #19 | ((decreas* or reduc* or discourag*) NEAR/2 (sedentary or inactiv*)):ti,ab,kw | 1120 |
| #20 | #16 OR #17 OR #18 OR #19 | 319229 |
| #21 | #6 AND #15 AND #20 | 7320 |
|  | **Cochrane reviews** | **23** |
|  | Cochrane protocols | 1 |
|  | Trials | 7295 |
|  | Editorials | 1 |

**Epistemonikos**

| **ID** | **Search** | **Hits** |
| --- | --- | --- |
| #1 | (advanced_title_en:(woman OR women OR girl* OR female) OR (advanced_title_en:(menstrua* OR menses OR menorrhagia OR menarche OR eumenorrh*) OR advanced_abstract_en:( menstrua* OR menses OR menorrhagia OR menarche OR eumenorrh*) |  |
| #2 | (advanced_title_en:(exercise* OR sport* OR “physical activity” OR “physical activities” OR “physical training” OR “physical education) OR advanced_abstract_en:( exercise* OR sport* OR “physical activity” OR “physical activities” OR “physical training” OR “physical education) |  |
| #3 | #1 AND #2 | **103** |

| **Database** | **Results** |
| --- | --- |
| Medline | 661 |
| Ovid EMCARE | 409 |
| AMED | 10 |
| CINAHL | 257 |
| Sport Discuss | 109 |
| Cochrane (SR’s) | 23 |
| Epistemonikos | 103 |
| **Total** | **1572** |
| Duplicates Endnote | 517 |
| Duplicates Raayan | 15 |
| **Final Total to Screen** | **1040** |
