# Supplementary material for: Interventions that support women, girls, and people who menstruate to participate in physical activity: a rapid overview of reviews
Source: BMC Public Health. 2026 Mar 27;26:1472. doi: 10.1186/s12889-026-27122-9 (PMC13147802; doi:10.1186/s12889-026-27122-9)
Supplement: Supplementary file 4 — Additional file 4: Websites of key third sector and government organisations A table detailing the websites of key third sector and government organisations. [file 12889_2026_27122_MOESM4_ESM.docx]

**Additional file 4: Websites of key third sector and government organisations**

| Google Advanced Search  <https://www.google.co.uk/advanced_search> |
| --- |
| NHS England  <https://www.england.nhs.uk/> |
| NHS Wales  <https://www.wales.nhs.uk/> |
| NHS Scotland  <https://www.careers.nhs.scot/careers/find-your-career/returning-to-practice/> |
| Health and Social Care Northern Ireland  <https://www.hscni.net/> |
| Welsh Government  <https://gov.wales/> |
| UK Government  <https://www.gov.uk/> |
| Scottish Government  <https://www.gov.scot/> |
| Northern Ireland Executive  <https://www.northernireland.gov.uk/> |
| World Health Organization  [www.who.int](http://www.who.int) |
| World Health Organization Institutional Repository for Information Sharing (IRIS)  <https://iris.who.int/> |
| Centers for Disease Control and Prevention  <https://www.cdc.gov/> |
| National Institute for Health and Care Excellence  <https://www.nice.org.uk/> |
| Public Health Wales  <https://phw.nhs.wales/> |
| UK Health Security Agency  <https://researchportal.ukhsa.gov.uk/> |
| Office for Health Improvement & Disparities  <https://www.gov.uk/government/organisations/office-for-health-improvement-and-disparities> |
| Public Health Scotland  <https://publichealthscotland.scot/> |
| Public Health Northern Ireland  <https://www.publichealth.hscni.net/> |
| Women in Sport  <https://womeninsport.org/> |
| Sport Wales  <https://www.sport.wales/> |
| Sport England  <https://www.sportengland.org/> |
| Sport Scotland  <https://sportscotland.org.uk/> |
| Sport Northern Ireland  <http://www.sportni.net/> |
| Youth Sport Trust  <https://www.youthsporttrust.org/> |
| Period Education UK  <https://www.periodeducation.org/> |
| Optimal Period  <https://optimalperiod.com/> |
| Bloody Brilliant  <https://bloodybrilliant.wales/> |
| Bloody Good Period  <https://www.bloodygoodperiod.com/> |
| Menstruation Research Network  <https://menstruationresearchnetwork.org.uk/> |
| Fair Treatment for the Women of Wales  <https://www.ftww.org.uk/> |
| Plan International UK  <https://plan-uk.org/resources> |
| Women Connect First  <https://womenconnectfirst.org.uk/> |
| Womankind Worldwide  <https://www.womankind.org.uk/> |
| UN Women  <https://www.unwomen.org/en> |
| Global Fund for Women  <https://www.globalfundforwomen.org/what-we-do/> |
| The National Alliance of Women’s Organisations (NAWO)  <https://nawo.org.uk/> |
| Women for Women International (Resources Library)  <https://www.womenforwomen.org/monitoring-evaluation-research-and-learning-resource-library?_gl=1*8bly34*_ga*MTQxNjkwNzE5OS4xNzEyMTUxNjM1*_ga_V8MFX11JQX*MTcxMjE1MTYzNS4xLjEuMTcxMjE1MTk5NC40Mi4wLjA> |
| International Alliance of Women  <https://womenalliance.org/> |
