# Supplementary material for: Interventions that support women, girls, and people who menstruate to participate in physical activity: a rapid overview of reviews
Source: BMC Public Health. 2026 Mar 27;26:1472. doi: 10.1186/s12889-026-27122-9 (PMC13147802; doi:10.1186/s12889-026-27122-9)
Supplement: Supplementary file 5 — Additional file 5: List of excluded reports Table of reports excluded on full text screening with the reason for exclusion. [file 12889_2026_27122_MOESM5_ESM.docx]

**Additional material 5: List of excluded reports**

| **Reports excluded on full-text screening** | **Reason for exclusion** |
| --- | --- |
| 1. AlSwayied et al. (2022). Assessing the acceptability and effectiveness of mobile-based physical activity interventions for midlife women during menopause: systematic review of the literature. *Journal of Medical Internet Research*; 10(12):e40271. <https://doi.org/10.2196/40271> | Wrong population  (menopausal and post-menopausal women) |
| 1. Arinze NA, McGarry JE. (2021). Identities and Relationships: Black and Latina Adolescent girls in Sport-Based Youth Development Programs. Child and Adolescent Social Work Journal. 38(4): 475-86. <https://doi.org/10.1007/s10560-021-00775-8> | Wrong review design  Not a SR |
| 1. Baird J, Cooper C, Margetts BM, et al. (2009) Workshop on ‘ Changing nutrition behaviour to improve maternal and fetal health ’ Changing health behaviour of young women from disadvantaged backgrounds : evidence from systematic reviews. Proceedings of the Nutrition Society (2009), 68, 195–204.   <https://doi.org/10.1017/S0029665109001050> | Wrong timeframe  Umbrella review with only one relevant SR that was published prior to 2008 |
| 1. Bland V, Sharma M. (2017). Physical activity interventions in African American women: A systematic review. Health Promot Perspect. 7(2): 52-9.   <https://doi.org/10.15171/hpp.2017.11> | Wrong review design  No critical appraisal was conducted |
| 1. Buelo AK, Kirk A, Lindsay RS, et al. (2019). Exploring the effectiveness of physical activity interventions in women with previous gestational diabetes: A systematic review of quantitative and qualitative studies. Prev Med Rep. 14: 100877.   <https://doi.org/10.1016/j.pmedr.2019.100877> | Wrong population  40% of the quantitative sample is postpartum mothers |
| 1. Chin HG. (2014). Physical activity in women: current guidelines and strategies for promoting compliance. Clin Obstet Gynecol. 57(3): 456-64.   https://doi.org/[10.1097/GRF.0000000000000045](https://doi.org/10.1097/grf.0000000000000045) | Wrong review design  Not a SR |
| 1. Cleland V, Granados A, Crawford D, et al. (2013). Effectiveness of interventions to promote physical activity among socioeconomically disadvantaged women: a systematic review and meta-analysis. Obes Rev. 14(3): 197-212.   https://doi.org/[10.1111/j.1467-789X.2012.01058.x](https://doi.org/10.1111/j.1467-789x.2012.01058.x) | Wrong population  More than 25% is over 51 (post-menopausal) |
| 1. Congello NC, Koniak-Griffin D. (2018). Review: Partner Support and Physical Activity among Mexican American Women. Ethn Dis. 28(4): 555-60.   <https://doi.org/10.18865/ed.28.4.555> | Wrong review design  No quality appraisal conducted |
| 1. Cotie LM, Prince SA, Elliott CG, et al. (2018). The effectiveness of eHealth interventions on physical activity and measures of obesity among working-age women: a systematic review and meta-analysis. Obes Rev. 19(10): 1340-58.   <https://doi.org/10.1111/obr.12700> | Wrong population  Includes women with chronic conditions |
| 1. Gagliardi AR, Morrison C, Anderson NN. (2022). The design and impact of culturally-safe community-based physical activity promotion for immigrant women: descriptive review. BMC Public Health. 22(1): 430.   <https://doi.org/10.1186/s12889-022-12828-3> | Wrong review design  A descriptive review with no quality assessment |
| 1. Gletsu M, Tovin M. (2010). African American women and physical activity. Physical Therapy Reviews. 15(5): 405-9.   <https://doi.org/10.1179/1743288X10Y.0000000011> | Wrong review design  No detailed critical appraisal |
| 1. Hull R, De Oliveira R, Zaidell L. (2018). An Ecological Approach to Exploring Physical Activity Interventions Aimed at Young UK-Based Females: A Narrative Systematic Review. Psychology. 9(14).   https://doi.org/10.4236/psych.2018.914161 | Wrong outcomes  No PA outcomes |
| 1. Jenkins F, Jenkins C, Gregoski MJ, et al. (2017). Interventions Promoting Physical Activity in African American Women: An Integrative Review. J Cardiovasc Nurs. 32(1): 22-9.   <https://doi.org/10.1097/jcn.0000000000000298> | Wrong population  More than 25% is over 51 (post-menopausal) |
| 1. Jiménez-Mérida MR, Romero-Saldaña M, Molina-Luque R, et al. (2021). Women-centred workplace health promotion interventions: a systematic review. Int Nurs Rev. 68(1): 90-8.   <https://doi.org/10.1111/inr.12637> | Wrong outcomes  No PA outcomes |
| 1. Joseph RP, Royse KE, Benitez TJ (2019) A Systematic Review of Electronic and Mobile Health (e- and mHealth) Physical Activity Interventions for African American and Hispanic Women. Journal of Physical Activity and Health. 16(3):230-239.   <https://doi.org/10.1123/jpah.2018-0103> | Wrong population  More than 25% is over 51 (post-menopausal) |
| 1. Juwono ID, Kun B, Demetrovics Z, et al. (2021). Mothers’ Physical Activity in the New Millennium: A Systematic Review of the Literature. Baltic Journal of Sport and Health Sciences. 4: 4-23.   <https://doi.org/10.33607/bjshs.v4i119.1015> | Wrong population  More than 25% are under 8 |
| 1. Laird Y, Fawkner S, Kelly P, et al. (2016). The role of social support on physical activity behaviour in adolescent girls: a systematic review and meta-analysis. Int J Behav Nutr Phys Act. 13: 79.   <https://doi.org/10.1186/s12966-016-0405-7> | Wrong intervention  Primarily studies of associations |
| 1. Osabi LA, van de Klundert J, Alhurishi SA, et al. (2023). A theory-informed systematic review to understand physical activity among women in Gulf Cooperation Council countries. BMC Public Health. 23(1): 1009.   <https://doi.org/10.1186/s12889-023-15725-5> | Not effectiveness review  Focus on factors and barriers and facilitators rather than effectiveness |
| 1. Peralta LR, Cotton WG, Dudley DA, et al. (2021). Group-based physical activity interventions for postpartum women with children aged 0-5 years old: a systematic review of randomized controlled trials. BMC Womens Health. 21(1): 435.   <https://doi.org/10.1186/s12905-021-01581-1> | Wrong population  Postpartum women |
| 1. Perez A, Fleury J, Keller C. (2010). Review of intervention studies promoting physical activity in Hispanic women. West J Nurs Res. 32(3): 341-62.   <https://doi.org/10.1177/0193945909351300> | Wrong review design  No quality appraisal conducted |
| 1. Prince S, Reed J, Martinello N, et al. (2016). Why do Adult Women Exercise? – A Systematic Review of Prospective Cohort Studies. Canadian Journal of Cardiology. 32(4): PS6-PS7.   <https://doi.org/10.1016/j.cjca.2016.02.020> | Conference abstract |
| 1. Prince SA, Reed JL, Martinello N, et al. (2016). Why are adult women physically active? A systematic review of prospective cohort studies to identify intrapersonal, social environmental and physical environmental determinants. Obes Rev. 17(10): 919-44.   <https://doi.org/10.1111/obr.12432> | Not effectiveness review  Focus on factors rather than effectiveness |
| 1. Sediva H, Cartwright T, Robertson C, et al. (2022). Behavior Change Techniques in Digital Health Interventions for Midlife Women: Systematic Review. JMIR Mhealth Uhealth. 10(11): e37234.   <https://doi.org/10.2196/37234> | Wrong population  More than 25% is over 51 (post-menopausal) |
| 1. Sharma M. (2008). Physical activity interventions in Hispanic American girls and women. Obes Rev. 9(6): 560-71.   <https://doi.org/10.1111/j.1467-789X.2008.00501.x> | Wrong review design  No quality appraisal conducted |
| 1. Standiford Brown A. (2009). Promoting physical activity amongst adolescent girls. Issues Compr Pediatr Nurs. 32(2): 49-64.   <https://doi.org/10.1080/01460860902737400> | Wrong review design  No quality appraisal conducted |
| 1. Tcymbal A, Demetriou Y, Kelso A, et al. (2020). Effects of the built environment on physical activity: a systematic review of longitudinal studies taking sex/gender into account. Environ Health Prev Med. 25(1): 75.   <https://doi.org/10.1186/s12199-020-00915-z> | Wrong population  Includes participants out of the relevant age range (8-51) |
| 1. Turner J, Clanchy K, Vincze L. (2023). Telehealth interventions for physical activity and exercise participation in postpartum women: A quantitative systematic review. Prev Med. 167: 107413.   <https://doi.org/10.1016/j.ypmed.2022.107413> | Wrong review design  No quality appraisal conducted |
| 1. Weber Buchholz S, Wilbur J, Halloway S, et al. (2013). Physical activity intervention studies and their relationship to body composition in healthy women. Annu Rev Nurs Res. 31: 71-142.   <https://doi.org/10.1891/0739-6686.31.71> | Wrong review design  No quality appraisal conducted |
| 1. White BM, Rochell JK, Warren JR. (2020). Promoting Cardiovascular Health for African American Women: An Integrative Review of Interventions. J Womens Health (Larchmt). 29(7): 952-70.   <https://doi.org/10.1089/jwh.2018.7580> | Wrong review design  No quality appraisal conducted |
| 1. Whitt-Glover MC, Brand DJ, Turner ME, et al. (2009). Increasing physical activity among African-American women and girls. Curr Sports Med Rep. 8(6): 318-24   <https://doi.org/10.1249/JSR.0b013e3181c27ade> | Wrong review design  No quality appraisal conducted and only one database searched |
| 1. Williams WM, Yeo S. (2016). Is Attitude a Key Factor to Consider When Designing Physical Activity Interventions for Black Adolescent Girls:A Review. Journal of Black Psychology. 42(1): 3-28.   <https://doi.org/10.1177/0095798414551790> | Wrong review design  No quality appraisal conducted |
