# Supplementary material for: Interventions that support women, girls, and people who menstruate to participate in physical activity: a rapid overview of reviews
Source: BMC Public Health. 2026 Mar 27;26:1472. doi: 10.1186/s12889-026-27122-9 (PMC13147802; doi:10.1186/s12889-026-27122-9)
Supplement: Supplementary file 6 — Additional file 6: Data extraction Data extraction table containing the characteristics and findings of included systematic reviews. [file 12889_2026_27122_MOESM6_ESM.docx]

**Additional file 6: Data extraction**

| **Citation** | **Review details** | **Included studies** | **Quality of the**  **primary research**  **Quality of the review** | **Findings** |
| --- | --- | --- | --- | --- |
| Allison et al. 2017  Is team sport the key to getting everybody active, every day? A systematic review of physical activity interventions aimed at increasing girls’ participation in team sport  <https://doi.org/10.3934/publichealth.2017.2.202> | Publication year of included studies  2008-2013  Review purpose  To review the evidence for the effectiveness of team sport interventions aimed at girls aged 11–18 years on team sport participation and wider physical activity outcomes  Included study designs  Mixed methods (n=3)  -With quantitative cross-sectional elements (surveys)  -Quantitative Pre-post design  Qualitative (n=1)  Outcomes of interest  Team sport participation, retention, trying new sports  -Self-report (surveys, attendance registers, registration forms) | Number of included studies  n=4 (of which one is qualitative), all grey literature reports  Interventions  Partnership working (n=1)  Grant funding (n=1)  Multicomponent (n=2), including:  Training for staff (n=2), Action plan (n=1), Grant funding (n=1), Partnership working (n=2), Resource provision (n=2), How-to guide (n=1), Gender-specific research insights (n=1), Merchandise (n=1)  No information about managing menstruation and the menstrual cycle was reported  *Focus:*  Not reported  *Intervention length:*  Not reported  *Follow-up:*  Not reported  Participants  Girls only (n=3)  Mixed (data separated for girls) (n=1)  Age  Between 11 – 25 years  Ethnicity  Not reported  Countries of interventions  UK (n=4): England (n=2), Scotland (n=2)  Settings  Community (n=2), School (n=1), Mixed (n=1) | Appraisal scale used for included studies  A custom tool based on CASP, NICE, University of Auckland, and CEBM tools  Appraisal rating of included studies  10/10 (n=1), 10/11 (n=1), 9/10 (n=1), 9/11 (n=1) Scores not interpreted  GRADE completed by systematic review authors  Not reported  Review appraisal score  6 out of 11 in the JBI checklist for systematic reviews and research syntheses | Main findings  *PA Participation*  “Fit for Girls” (multicomponent intervention): 59.9% of PE staff said it helped to **increase girls’ participation in PE, sport and PA**, and 47.5% said that it helped to increase participation among low-active girls.  **No increased participation in extracurricular sport**: 60.8% of girls did not take part in an extracurricular sport or PA during the previous week, compared to 47.5% in the previous year‘s evaluation.  There was an **increase from 39% to 45% of girls participating in sports clubs outside of school**.  “Girls on the Move” (grant funding intervention): It is estimated that around 1,800 girls took part in activities provided**. 53% of girls had high attendance rates** and were involved from the beginning to end of the project. **25% of girls had low attendance** (less than 25% of sessions), with some girls only attending one session before dropping out.  *Retention*  “Sportivate” (partnership working intervention): There was a **2.2% decrease in female participants from year 2-3**, but **females were just as likely to be retained as males** once they had found an activity which suited them. There was a **7.1% decrease in the level of female participation aged 16 when compared to aged 14**.  *Trying new sports*  “Fit for Girls” (multicomponent intervention): **62.2% of girls took part in an activity they had not tried before**. 49.2% took part in girls-only activities. 37.3% took part in an activity outside of school after trying it at school.  Additional findings  *Success of new clubs/sports*  “Fit for Girls” (multicomponent intervention): Fewer girls agreed that boys and girls should do PE separately (30.8% compared with 37% over the course of the intervention). For many of the girls, being able to opt in to girls-only activities was really important in terms of their own confidence and enjoyment within the PE class and, consequently, their levels of participation. Less active girls were more likely to prefer girls only classes than more active girls (33.6% compared with 28.8%).  *Perceived barriers*  “Fit for Girls” (multicomponent intervention): “Lack of time” increased from 27.1% to 41.4% of participants reporting it as a barrier post-intervention. “Cost of activities” as a barrier increased by 10%. |
| Amiri Farahani et al. 2015  Community-based physical activity interventions among women: a systematic review  <http://dx.doi.org/10.1136/bmjopen-2014-007210> | Publication year of included studies  2003-2010  Review purpose  To investigate strategies for promoting physical activity among women aged 18–65 years, and conducted with community-based  approaches  Included study designs  RCTs (n=3)  Non-randomised studies (n=6)  Outcomes of interest  PA participation  -Self-report (questionnaires, recall instruments);  -Objective (pedometers) | Number of included studies  n=9  Interventions  Multicomponent (n=9)  No information about managing menstruation and the menstrual cycle was reported  *Focus:*  PA only (n=5)  PA and diet (n=4)  *Intervention length:*  8 weeks (n=2)  10 weeks (n=1)  12 weeks (n=1)  4 months (n=1)  12 months (n=3)  Mixed (12 weeks and 6 months) (n=1)  *Follow-up:*  Immediate post-intervention only (n=7)  12 months follow-up (n=2)  Participants  Women  Ethnicity  Not reported  Age  Between 18 - 65 years  Mean age or range under 51 (n=7, 78%)  Over 51 (n=2, 22%)  Countries of interventions  USA (n=7); Australia (n=1); Iran (n=1)  Settings  Community (community-based PA interventions involve community members and leaders from various settings and organisations (i.e.. at any of the four ecological levels) in the design, implementation and evaluation of a PA intervention) | Appraisal scale used for included studies  The appraisal is composed of seven scales including Delphi List, PEDro, Maastricht, Maastricht-Amsterdam List, Bizzini, vanTulder and Jadad. The appraisal was compiled in a set of 39 items by Olivo et al. (2008) where the items were divided into five categories: patient selection, blinding, interventions, outcomes and statistics  Appraisal rating of included studies  High (score 0.51 to 1) (n=5)  Low (score 0 to 0.5) (n=4)  GRADE completed by systematic review authors  Not reported  Review appraisal score  7 out of 11 in the JBI checklist for systematic reviews and research syntheses | Main findings  Results showed that most of the articles were limited or had inconclusive evidence of an interventions’ effect.  Seven studies reported a positive intervention effect (77.7%), and in 4 of these studies statistical significance was achieved (44.45%).  Significant results ranged from an increase of 2.07 days per week in doing aerobic exercise to a 10.4% increase in participation in regular PA (at least 30 min of moderate intensity PA for at least 5 days a week, or at least 20 min of vigorous PA for at least 3 days a week). |
| Barnes et al. 2018  Effectiveness of mother and daughter interventions targeting physical activity, fitness, nutrition and adiposity: a systematic review  <https://doi.org/10.1016/j.ypmed.2017.12.033> | Publication year of included studies  1990-2015  Review purpose  To assess the effectiveness of lifestyle behavior change interventions targeting mothers and their daughters on participant adiposity, physical activity, fitness and dietary outcomes  Included study designs  RCTs (n=7)  Pseudo-randomized  controlled trial (n=1)  Non-randomised controlled trial (n=1)  Pre-post test trial (n=5)  Outcomes of interest  PA or fitness-related outcomes  -Self-report  -Objective (accelerometry and/or pedometry) | Number of included studies  n=14 studies across 16 reports  Interventions  Community-based intervention for mothers and daughters that targeted physical activity, fitness, nutrition, or adiposity.  No information about managing menstruation and the menstrual cycle was reported  *Focus:*  PA and fitness (n=6)  PA and diet (n=5)  Diet only (n=2)  PA, fitness and diet (n=1)  *Intervention length:*  <12 weeks (n=6)  12 weeks (n=6)  16 weeks (n=1)  6 months (n=1)  *Follow-up:*  Immediate post-intervention only (n=9)  20 weeks (n=1)  5 months (n=1)  6 months (n=3)  Participants  Mothers and daughters  Age  Daughters: Between 8 - 19 years (n=10, 71%)  Studies including participants younger than 8 years (included age ranges: 5-12, 7-13, 7-12, 6-9) (n=4)  Mothers: Mean age ranged between 32 and 45.2 years (n=12)  Age not reported (n=2)  One study also included grandmothers (Age range 50-70 years)  Ethnicity  Not reported  Countries of interventions  USA (n=11); Iran (n=2); Australia (n=1)  Settings  Community (n=9 out of which University (n=3), school (n=3), tutoring (n=2), unspecified (n=1))  Home (n=2)  Summer camp (n=2)  Church (n=1) | Appraisal scale used for included studies  Nine-item tool adapted from Consolidated Standards  of Reporting Trials (CONSORT) statement and previously used quality criteria  Appraisal rating of included studies  Studies meeting the criteria for each of 9 domains:  Randomisation clear (n=1)  Validated outcome measure (n=9)  Assessor blinding (n=0)  ITT (n=3)  Analyses adjusted for covariates (n=6)  Power calculation provided (n=0)  Baseline outcome data presented by group (n=10)  Dropout ≤20% for ≤6 m follow-up and ≤30% for> 6 m (n=8-9 (different for girls and mothers))  Summary results estimated effect size +precision (n=5)  GRADE completed by systematic review authors  Not reported  Review appraisal score  7 out of 11 in the JBI checklist for systematic reviews and research syntheses | Main findings  *PA: Significant group-by-time effect (favoring the intervention group)*  *RCTs (Out of 7 RCTs 5 measured PA outcomes)*  Mothers (Out of 5 only 4 reported PA outcomes):  Post test: **Two studies** (2 outcomes measures across 10) **reported significant effect for PA** measured via self-report PA survey and steps/day (combined across mother and daughters).  Follow up: **One study** (1 outcome measure across 7) **reported significant effect for PA** measured via Vigorous PA (%)  Daughters (Out of 5 all 5 reported PA outcomes):  Post test: **One study** (1 outcome measure across 9) **reported significant effect for PA** measured via MVPA mins.  Follow up: **No study** (0 outcome measure across 6) **reported significant effect for PA**  *Non RCTs (Out of 7 non RCTs 3 measured PA outcomes)*  None of the studies investigated group-by-time effect post-test or follow-up.  *PA: Significant within group change*  *RCTs (Out of 7 RCTs 5 measured PA outcomes)*  Mothers (Out of 5 only 4 reported PA outcomes):  Post test: **Two studies** (3 outcomes measures across 10) **reported significant effect for PA** measured via Vigorous PA (%), Self-report MVPA, Aerobic (days/week).  Follow up: **No study** (0 outcome measure across 7) **reported significant effect for PA**  Daughters (Out of 5 only 4 reported PA outcomes):  Post test: **Two studies** (3 outcomes measures across 9) **reported significant effect for PA** measured via Mean counts/min, Light PA (%), Aerobic (days/week).  Follow up: One study (1 outcome measure across 6) reported significant effect for PA measured via Mean counts/min  *Non RCTs (Out of 7 non RCTs 3 measured PA outcomes)*  Mothers (Out of 3 only 2 reported PA outcomes):  Post test: No study (0 outcome measure across 2) reported significant effect for PA (days/week)  Follow up: Only one study conducted follow up assessment, the results of which were not significant (PA days/week)  Daughters (Out of 3 only 1 reported PA outcomes (outcomes were unclear for 2 non RCTs)):  Post test: No study (0 outcome measure across 1) reported significant effect for PA (days/week)  Follow up: Only one study conducted follow up assessment, the results of which were not significant (PA days/week)  Additional findings  Successful interventions aiming to improve physical activity, fitness, nutrition and adiposity were multi-component, were designed to allow mothers and daughters to participate together and had multiple sessions offered over the course of the intervention. |
| Biddle et al. 2014b  The effectiveness of interventions to increase physical activity among young girls: a meta analysis  <http://dx.doi.org/10.1016/j.ypmed.2014.02.009>  Biddle et al. 2014a  Corrigendum to “The effectiveness of interventions to increase physical activity among young girls: A meta-analysis”  <http://dx.doi.org/10.1016/j.ypmed.2014.07.014> | Publication year of included studies  1997-2013  Review purpose  To quantify the effect of physical activity interventions for pre-adolescent girls by including intervention studies that provided results for girls separately  Included study designs  RCTs (n=16) (including: pilot (n=2), group (n=1) and parallel group (n=1) designs)  Controlled Study (n=5)  Treatment control repeated measures (n=1)  Outcomes of interest  PA behaviour  -Self-report (n=5)  -Objective (n=13)  -Combination of objective and self-report (n=4) | Number of included studies  n=22  Interventions  Educational (n=9)  Environmental (n=4)  Multicomponent (n=9)  No information about managing menstruation and the menstrual cycle was reported  *Focus:*  PA only (n=12)  PA and diet (n=7)  Obesity related behaviours (n=3)  *Intervention length:*  ≤ 12 weeks (n=8)  > 12 weeks (n=14)  *Follow-up:*  Immediate post-intervention only (n=14)  Studies with follow-up (n=8) (follow-up period not specified)  Participants  Pre-adolescents  Girls only (n=6)  Mixed (data separated for girls) (n=16)  Age  Between 8 – 12 years (n=19, 86%)  Younger than 8 years (n=3, 14%)  Ethnicity  Not reported  Countries of interventions  USA (n=14); UK (n=2); Norway (n=1); Denmark (n=1); Cyprus (n=1); Greece (n=1); Australia (n=1); Belgium (n=1)  Settings  School (n=10)  School and outside of school (n=7)  Community (n=3)  Community and Family (n=2) | Appraisal scale used for included studies  Cochrane Collaboration tool for Assessing Risk of Bias: 7 domains scoring either high, low or unclear  Delphi Score:  -1 = High risk (>6)  0 = unclear risk (4-6)  1 = low risk (<3)  Appraisal rating of included studies  High Delphi score (n=7)  Moderate Delphi score (n=9)  Low Delphi score (n=6)  GRADE completed by systematic review authors  Not reported  Review appraisal score  7 out of 11 in the JBI checklist for systematic reviews and research syntheses | Main findings  There was a significant small positive treatment effect (k=22, g=0.314, 95% CI (0.112, 0.516), p<0.001) for experimental groups participating in physical activity interventions.  The differential score between treatment and control groups indicated that there was approximately one third of a standard deviation or the equivalent of 12.17% more physical activity for girls participating in the experimental conditions.  Heterogeneity: There was significant between-study variance (Q_T_=346.37, p<0.001, τ^2^=0.199, I^2^=93.94) and that a large portion of variance could be explained by subgroup analyses.  Additional findings  *Subgroup analysis:*  *Sample characteristics (girls only vs mixed):*  Significant between subgroup differences (Q_B_=7.522, p<0.001) existed when studies developed interventions for girls only (k=6, g=0.774, 95% CI (0.396, 1.152)) compared to studies involving both boys and girls (k=16, g=0.174, 95% CI (−0.028, 0.377)).  *Intervention type (within subgroup differences)*:  Educational interventions (k=9, g=0.414, 95% CI (0.070, 0.759), p<0.01) Heterogeneity (τ^2^=0.067, I^2^=82.43)  Environmental (k=4, g= -0.301, 95% CI (−0.795, 0.194), p>0.01) Heterogeneity (τ^2^=1.174, I^2^=98.11)  Multicomponent (k=9, g=0.503, 95% CI (0.172, 0.833), p<0.01) Heterogeneity (τ^2^=0.174, I^2^=93.17)  *Intervention length (within subgroup differences*):  ≤ 12 weeks (k=8, g=0.636, 95% CI (0.290, 0.983), p<0.01) Heterogeneity (τ^2^=0.026, I^2^=40.94)  > 12 weeks (k=14, g=0.155, 95% CI (−0.088, 0.398), p>0.01) Heterogeneity (τ^2^=0.214, I^2^=95.76)  *Study quality* *(within subgroup differences)*  High (k=7, g=0.588, 95% CI (0.200, 0.976), p<0.01) Heterogeneity (τ^2^=0.348, I^2^=94.15)  Low (k=6, g= -0.170, 95% CI (-0.573, 0.233), p>0.01) Heterogeneity (τ^2^=0.404, I^2^=97.16)  Moderate (k=9, g=0.448, 95% CI (0.116, 0.781), p<0.01) Heterogeneity (τ^2^=0.049, I^2^=79.01)  *Theoretical approach (within subgroup differences)*  Atheoretical (k=10, g=0.526, 95% CI (0.235, 0.817), p<0.01) Heterogeneity (τ^2^=0.154, I^2^=91.82)  Theoretical (k=12, g= 0.120, 95% CI (-0.158, 0.399), p>0.01) Heterogeneity (τ^2^=0.232, I^2^=94.38) |
| Brennan et al. 2021  A systematic review of the intervention characteristics, and behavior change theory and techniques used in mother-daughter interventions targeting physical activity  <https://doi.org/10.1016/j.ypmed.2021.106764> | Publication year of included studies  2001-2019  Review purpose  To examine the intervention characteristics, and behavior change theory and techniques used in interventions that promote physical activity for mothers and daughters  Included study designs  RCTs (n=6)  Non-RCTs (n=5)  Outcomes of interest  PA levels  -Self-report (n=4)  -Objective (Device-based measures (pedometers) (n=5))  -Combination of device-based (pedometers) and self-report (n=2) | Number of included studies  n=11 studies across 16 reports  Interventions  The 11 studies investigated 14 intervention arms, as three studies compared two interventions with/without a control group  Mother and daughter interventions targeting PA levels  No information about managing menstruation and the menstrual cycle was reported  *Focus (based on n=11* s*tudies)*  PA only (n=8)  PA and diet (n=3)  *Intervention length (based on n=14 intervention arms):*  ≤3 months (n=11)  >3 to ≤12 months (n=3)  *Follow-up (based on n=14 intervention arms):*  Immediate post-intervention only (n=10)  Studies with follow-up (n=4) (follow-up period not specified)  Participants (based on n=14 intervention arms)  Mothers and daughters  -Intervention delivered to mothers and daughters together (n=11)  -Interventions delivered separately (n=2)  -Interventions delivered just to daughters (n=1)  Age  Daughters: Between 7-17 years (Mean age was younger than 8 years in 1 intervention group from 1 study)  Mothers: Between 28-50 years  Ethnicity  Not reported  Countries of interventions (n=11 studies)  USA (n=9); Australia (n=2)  Settings (based on n=14 intervention arms)  Community (n=9)  After school (n=2)  Home (n=2)  Online (n=1) | Appraisal scale used for included studies  Cochrane RoB tool for RCTs  ROBINS-I tool for non-RCTs  Appraisal rating of included studies  High risk of bias (n=6, RCTs)  Critical risk of bias (n=5, non-RCTs)  GRADE completed by systematic review authors  Not reported  Review appraisal score  8 out of 11 in the JBI checklist for systematic reviews and research syntheses | Main findings  *RCTs*  For the nine intervention groups targeting **daughters' PA** as part of an RCT, there was a **significant increase in PA** from baseline to post intervention in **five groups** and a between-group change for **one group**  Four of eight intervention groups targeting **mothers' PA levels** as part of an RCT reported an **increase in PA levels** either from baseline to post-intervention (n = 3) or a between group change (n = 1)  *Non-RCTs*  For the non-RCT studies, one study showed a significant increase in PA levels for daughters and mothers and another for daughters only.  Two non-RCTs did not perform statistical tests due to small sample sizes but reported mean changes in PA from baseline to follow-up. Both of these studies reported a mean increase in PA levels for both daughters and mothers  Additional findings  The most frequently reported behaviour change techniques (i.e., demonstration of behaviour, instruction on how to perform the behaviour, behavioural practice/rehearsal, credible source) had a potential effectiveness ratio of at least 70% for daughters but for mothers it was typically lower (<60%). However, the behaviour change techniques with the highest potential effectiveness ratio were not the most frequently observed, with goal setting (behaviour) and information on the health consequences of the behaviour having ratios of 80% and 100% respectively. Other self-regulatory related techniques such as self-monitoring and problem-solving were also identified in at least 70% of promising interventions. |
| Camacho-Minano et al. 2011  Interventions to promote physical activity among young and adolescent girls: a systematic review  <https://doi.org/10.1093/her/cyr040> | Publication year of included studies  2003-2010  Review purpose  To conduct a systematic review to describe the available evidence of PA interventions that targeted both young and adolescent girls  Included study designs  RCTs (n=19)  Quasi-experimental (n=2)  Outcomes of interest  PA outcomes (defined as PA levels (n=9),  PA behaviour (n=11)  -Self-report (n=7)  -Objective (accelerometers, pedometers, heart rate monitors and cardiovascular fitness tests) (n=14) | Number of included studies  n=21 studies across 29 reports  Interventions  Educational and multicomponent but interventions are not formally grouped. Main grouping is based on the setting  No information about managing menstruation and the menstrual cycle was reported  *Focus*:  PA only (n=18)  PA and diet (n=3)  *Intervention length:*  1 week (n=1)  12 weeks (n=7)  4 months to 1 year (n=7)  3 years (n=1)  *Follow-up:*  Immediate post-intervention only (n=21)  Participants  Young (elementary school aged) and adolescent girls (secondary school aged, including middle and high-school)  Age  Young girls between 5-11 years (n=6)  Adolescent girls between 12-18 years (n=15)  Mean age was over 8 years in all studies  Ethnicity  Five of the studies conducted in the USA specifically aimed at ethnic minority groups, such as African American or Hispanic  Countries of interventions  USA (n=17), UK (n=1), Australia (n=2), Iran (n=1)  Settings  School (n=17) out of which non-curricular (after school programme) (n=5) and physical education specific (n=8)  Community (n=3) out of which Girl Scout troops (n=2) and summer camp;  Primary care (n=1) | Appraisal scale used for included studies  Checklist criteria were derived from previously used quality criteria and consisted of 11 criteria symbolizing the quality of the intervention based on the study’s internal validity and analysis.  A formal quality score for each study was completed on an 11-point scale by assigning the value of ‘1’ (yes) or ‘0’ (no, unclear, insufficiently described or non-applicable) to each of the items listed. Each criteria had the same value or weight, the sum of which was used as a methodological quality score and was calculated as a percentage of the maximum obtainable score.  Appraisal rating of included studies  High methodological quality (score >58%) (n=12)  Low methodological quality (score <58%) (n=9)  GRADE completed by systematic review authors  Not reported  Review appraisal score  6 out of 11 in the JBI checklist for systematic reviews and research syntheses | Main findings  *Effective interventions*  Findings from the PA intervention studies were **mixed with regard to their effectiveness***.* Of the 12 studies with an acceptable methodological quality, 5 failed to increase girls’ PA and although 7 were successful, in some cases, their results were modest.  Ten of the 21 studies reported a favorable intervention effect upon PA outcomes.   - Seven of which were rated as having a high methodological quality. Five of these interventions targeted specifically adolescent girls and two targeted young girls. - Three studies also reported positive results between-group effects on PA and/or CV fitness outcomes but were of low methodological quality. These focused on adolescent girls (middle school and high school).   *Ineffective interventions*  Five studies rated as having a high methodological quality reported a non-effective result upon PA outcomes.   - Two were aimed at adolescents and were based on a girls-only PE class and three targeted elementary school-aged girls.   The remaining six ineffective PA interventions were studies rated as low quality.   - Three of them targeted adolescent girls. |
| Houle et al. 2020  Effects of extra-curricular physical activity programs on high-school girls: a systematic review  <https://doi.org/10.1101/2020.04.25.20079780> | Publication year of included studies  2000-2018  Review purpose  To assess the efficacy of ECPAP specifically targeting adolescent girls  Included study designs  Cluster RCT (n=2)  Pre-test / Post-test quasi-experimental pilot (n=1)  Pre-post test study (n=1)  Outcomes of interest  PA levels (MVPA)  -Objective (Accelerometry) (n=4)  Sedentary activities  -Self-report  -Objective (Accelerometry) (n=2) | Number of included studies  n=17 (out of 17 included studies only 4 reported on PA outcomes and are extracted below)  Interventions  PA programme (n=2)  -Dance Programme (n=2)  Multi-approach programme (n=2)  -All included some discussion either one-to one or groups (health, PA, nutrition, empowerment and skill development)  -Some included a motivational component  -Monthly newsletters  -Text messages  No information about managing menstruation and the menstrual cycle was reported  *Focus:*  PA only (n=2)  PA and diet (n=2)  *Intervention length:*  Ranging between 8 weeks and 12 months  ≤ 12 weeks (n=1)  > 12 weeks (n=3)  *Follow-up:*  Not reported  Participants  Adolescent girls  Age  Between 11-17 years  Ethnicity  Not reported  Countries of interventions  UK (n=2), USA (n=1), Australia (n=1)  Settings  Schools (extracurricular) | Appraisal scale used for included studies  Downs and Black 27-item checklist – modified to improve scoring system  Scoring system was percentage of criteria properly fulfilled - The scoring was used in combination with the signs indicating positive,  negative or neutral findings  Appraisal rating of included studies  *Physical activity levels* *(n=4)*  46%, 64%, 68%, 86%  *Sedentary activities (n=2)*  64-68%, 86%  GRADE completed by systematic review authors  Not reported  Review appraisal score  6 out of 11 in the JBI checklist for systematic reviews and research syntheses | Main findings  Only four of the studies reported physical activity levels   - Between study heterogeneity makes impossible the comparison of the effect sizes on mean daily MVPA - **No intervention increased in mean daily MVPA minutes when averaged over several days**. - Martin and Fairclough (2008) observed a significant increase in MVPA of 19.3 ± 1.7 min (p<0.05) during their enhanced dance classes when compared to pre-intervention classes. - There was no significant difference in change in mean daily MVPA between the “Girls Bristol Dance Project” intervention and control group (Jago et al. 2015). - There was no significant difference in change in mean daily MVPA between the “NEAT Girls” intervention and control group (Lubans et al 2014) - The results were non-significant for the “Girls on the move” project (Robbins et al., 2012).   Additional findings  Two studies (across three reports) reported on sedentary activities   - The “NEAT Girls program” reduced significantly self-reported screen time, especially recreational computer use, and the sum of sedentary activities, with an adjusted median difference (interquartile) of -30.7 (-62.4 to -1.06) min/d, -26.0 (-46.9 to -5.1) min/d, and -56.4 (-110.1 to -2.7) min/d, respectively (Dewar et al., 2014; Lubans et al., 2012). - However, the objective measure of time spent sedentary using accelerometry did not show differences between the intervention and control group neither for “NEAT Girls” nor for “Girls Bristol Dance Project” (Dewar et al., 2014; Jago et al., 2015). |
| Kelly et al. 2024  A review of the impact of sporting role model-led interventions on physical activity and sport participation of female youth  <https://doi.org/10.1123/wspaj.2023-0010> | Publication year of included studies  Peer reviewed study report: 2012  Grey literature: Not reported  Review purpose  To identify and describe Sporting Role Model -led interventions that target PA and/or sport participation levels among female youth to determine  (a) the design of the program  (b) the impact on sport participation/PA, and  (c) provide recommendations for future Sporting Role Model programs in research, policy, and practice  Included study designs  Not reported  Outcomes of interest  *Peer reviewed paper:* PE attendance  *Grey literature:*  Not reported | Number of included studies  n=1 Peer reviewed study report  n=15 Grey literature  Interventions  ***Peer reviewed study report***  “Sky Living for Sports”: All schools were funded for between 2 and 3 months for 3 years to complete a variety of novel (e.g. skiing, judo, skateboarding, and orienteering) activities within school time  “Changing Lives” (Intervention): In addition to “Sky Living for Sport”, an elite sports role model visited the schools once per year. Sporting Role Models were world-class elite athletes selected based on capability to interact with adolescents by sharing their own struggles with adversity and how they overcame them  No information about managing menstruation and the menstrual cycle was reported.  *Intervention length:*  Sporting Role model visits once per year  *Follow-up:*  Not reported  ***Grey literature***  Sporting Role Models: Olympians/ Paralympians (n=5); Intercounty Gaelic Games players (n=3), High-profile, elite, or professional athletes (n=7)  No information about managing menstruation and the menstrual cycle was reported.  *Intervention length:*  One-off visit/event (n=10), Multiple visits/events (n=5)  *Follow-up:*  Not reported  Participants  *Peer reviewed paper:* Adolescents  *Grey literature:* Girls only (n=9), Mixed (n=6)  Age  *Peer reviewed paper:* Between 11-16 years  *Grey literature:* Not reported  Ethnicity  Not reported  Countries of interventions  *Peer reviewed paper:* UK (n=1)  *Grey literature:* Australia (n=7), Ireland (n=4), England (n=2), USA (n=1), New Zealand (n=1)  Settings  *Peer reviewed paper:* School  *Grey literature:* School (n=4), online (n=1), clubs/teams (n=2), Mixed (n=4), Juvenile justice centre (n=1), Koori community (n=1), Not reported (n=2) | Appraisal scale used for included studies  Downs and Black 27-item checklist  A score was allocated to each article between 0 and 32. Using the framework outlined by Hooper et al. (2008), the quality of each intervention was established using the following scale:  Excellent (26-28)  Good (20-25)  Fair (15-19)  Poor (≤14)  Appraisal rating of included studies  Peer reviewed article (n=1) Poor quality  Grey literature (n=15) Poor quality  GRADE completed by systematic review authors  Not reported  Review appraisal score  7 out of 11 in the JBI checklist for systematic reviews and research syntheses | Main findings  *Peer reviewed paper:*  Overall, there were no significant differences in PE attendance between the groups (Sky Living for Sports (3.6–4.2) and Changing Lives (3.5–4.1; p > 0.05))  *Grey literature:*  Unpublished/unavailable evaluations have been completed by the five Irish programs and one  Australian program. Two of the programs have published evaluation reports:   - “Football Association Players Ambassador Programme” case study: The program re-enthused female participation in soccer, inspired girls to believe they could play, delivered meaningful life lessons, and increased interest in the elite women’s game based on data collected on girls who attended the elite player visits, the elite athletes leading the program, and the organizers who facilitated the visits - “Sporting Champions”: Evidence of SRM impact was reported through self-report data collected from youth groups who interacted with the SRM and compared this with a control group of youths who did not interact with an elite SRM. The evaluation offered several recommendations including enhanced targeting of female participation, adjusting the questionnaire to identify what makes the athlete inspiring and motivating, including an additional focus group on exit route strategies from sport (to sustain participation after school), and the potential for further research in using sporting champions to retain participation in sport. |
| Madden et al. 2020  The effect of workplace lifestyle programmes on diet, physical activity, and weight-related outcomes for working women: A systematic review using the TIDieR checklist  <https://doi.org/10.1111/obr.13027> | Publication year of included studies  2010-2016  Review purpose  To identify the intervention characteristics of lifestyle programmes or organisational policy changes in the workplace associated  with improved diet, PA, or weight-related outcomes for working women  Included study designs  RCT (n=8)  Cluster RCT (n=2)  Controlled before and after (n=5)  Quasi-experimental (n=4, including 2 pilot studies)  Pilot study (n=1)  Outcomes of interest  PA outcomes  -Self-report  -Objective  (Steps, MET, oxygen consumption (VO_2_ peak/max), leisure activity score, MVPA, sit time, sit-to-stand transitions, aerobic minutes, meeting PA guidelines. Measured through self-reports, accelerometer, pedometer, heart rate monitor) | Number of included studies  n=20 studies across 23 reports  Interventions  The 20 studies investigated 26 intervention arms  Exercise (n=5)  Interrupted sitting (n=1)  Multicomponent (n=14), including:   - Education (n=12), Goal-setting (n=9), Exercise (n=7), Peer support (n=5), Incentives (n=3), Self-monitoring (n=3), Counselling (n=2), Health assessment (n=2), Self-efficacy (n=3), Weigh-in sessions (n=2), Cognitive restructuring (n=1), Environment scan (n=1), Feedback (n=1), Goal monitoring (n=1), Motivational interviewing (n=1), Overcoming barriers (n=1), Problem solving (n=1), Prevention (n=1), Relapse prevention (n=1), Skill building (n=1), Basic principles of behaviour modification (n=1)   No information about managing menstruation and the menstrual cycle was reported.  *Focus*  PA-only (n=8, 12 arms)  PA and diet (n=12, 14 arms)  *Intervention length (based on n=26 intervention arms):*  Ranging between 2.5 days to 3 years  Short duration (≤3 months) (n=12)  Medium duration (>3 months) (n=8)  Long duration (≥12 months) (n=6)  *Follow-up:*  Immediate post-intervention only (n=15)  Studies with follow-up (n=5) (Ranging from 20 weeks to 5 years)  Participants  Working women  Women only (n=10)  Mixed (n=10)  Age  Mean age ranged between 33.2±7.8 and 48.77+9.27 years (n=16)  Age not reported (n=4)  Ethnicity  Not reported  Countries of interventions  USA (n=9), Brazil (n=2), Iran (n=1),  Tunisia (n=1), Norway (n=1), Ireland (n=1), Sweden (n=1), Japan (n=1), Germany (n=1), Singapore (n=1), Netherlands (n=1)  Settings  Workplace (conducted at or facilitated by) (n=20 out of which outside working hours (n=9), during work time and leisure time (n=4), during paid work time (n=4), during paid work time and outside working hours (n=1)) | Appraisal scale used for included studies  Cochrane RoB 2.0 tool for RCTs  ROBINS-I tool for other study types  Appraisal rating of included studies  RoB 2.0:  Some concerns (n=4)  High risk of bias (n=6)  ROBINS-I:  Moderate risk of bias (n=2)  Serious risk of bias (n=5)  Critical risk of bias (n=3)  GRADE completed by systematic review authors  Not reported  Review appraisal score  8 out of 11 in the JBI checklist for systematic reviews and research syntheses | Main findings  *PA interventions with PA outcomes (n=10)*  At least one PA effect (n=7), no effect (n=3)   - Mean steps/day: effect (n=1), no effect (n=2), mixed (n=2) - Moderate steps/day: effect (n=1), no effect (n=2) - Weekly Leisure Activity Score: effect (n=2) - MVPA accelerometer: mixed (n=2) - Total accelerometer counts: mixed (n=2) - Workday sit time: effect (n=1) - Workday average sit time (hours): effect (n=1) - Workday sit-to-stand transitions: effect (n=1) - MET mins/week: mixed (n=1) - VO_2_ peak: effect (n=2) - VO_2_ max: effect (n=1), no effect (n=1)   *PA and diet interventions with PA outcomes (n=7)*  At least one PA effect (n=5), no effect (n=2)   - Mean steps/day: no effect (n=1) - aerobic minutes/day: effect (n=1) - Meeting PA guidelines: mixed (n=1) - Load-bearing MVPA/week: effect (n=1) - Load-bearing MVPA minutes: effect (n=1) - VO_2_ max: effect (n=2), no effect (n=2)   *All interventions reporting attrition (n=16)*  Low (n=4), medium (n=6), high (n=6)  *All interventions reporting adherence (n=12)*  Low (n=1), medium/high (n=11)  Additional findings (features of interventions)  *PA interventions with PA outcomes (n=10)*  Interventions that reported **improved PA outcomes** included a group format (n=6), in-person mode of delivery (n=7), a short to medium duration (n=7), delivery by non-HCPs (n=4), medium to high levels of adherence (n=5).  Interventions that reported **no effect** included technology (e.g. treadmill or Nintendo Wii^TM^) as their main component (n=3), lasted less than or equal to 3 months (n=3), used an individual mode of delivery (n=2), did not incorporate tailoring (n=2) or named theory (n=2).  *PA and diet interventions with PA outcomes (n=7)*  Interventions that reported **improved PA outcomes** were delivered by non-HCPs or a combination of non-HCPs and HCPs (n=4), included a group or combined individual and group format (n=4), used an in-person mode of delivery (n=5), lasted a medium to long duration (n=4), incorporated tailoring (n=4).  Interventions that reported **no effect** on lasted a medium to long duration (n=2) and were delivered in-person (n=2). |
| Matheson et al. 2023  A systematic review and meta-analysis of interventions that target the intersection of body image and movement among girls and women  <https://doi.org/10.1080/1750984X.2023.2258379> | Publication year of included studies  2007-2021  Review purpose  To examine interventions targeting the intersection of body image and movement experiences among girls and women  Included study designs  RCTs (n=2)  Cluster RCTs (n=2)  Outcomes of interest  Movement behaviour (Movement is broadly defined as the changing of your physical position  (Caspersen et al. 1985). Matheson et al. (2023) refer to movement or movement-based activities, as  it is more inclusive, holistic, and representative of the full spectrum of activities that  can foster and deepen one’s relationship with their body)  -Self-report  -Objective (number of steps walked) | Number of included studies  n=31 (out of 31 included studies only 4 reported on Movement behaviour outcomes and are extracted below)  Interventions  Unimodal (n=1) (hatha yoga)  Mulitmodal (n=3), including: physical activity intervention (n=1), Healthy me (strengths-based approach to enhance positive body image) (n=1), Healthy body image program (n=1)  No information about managing menstruation and the menstrual cycle was reported.  *Focus:*  Movement-based (n=2)  Body Image Based (n=2)  *Intervention length:*  4 weeks + recap session 3 months after the programme (n=1)  Not reported (n=3)  *Follow-up:*  Immediate post-intervention only (n=4)  Participants  Girls and women (mixed samples were considered for inclusion, but not reported whether the included studies contained girls or women only or male participants too)  Age  Between 12-17 years (n=2)  Between 0-11 years (Mean age 8.8 years)(n=1)  >35 years (Mean age 49.3 years) (n=1)  Ethnicity  Not reported  Countries of interventions  UK & Ireland (n=1)  Canada (n=1)  Australia (n=1)  Norway (n=1)  Settings  Not reported | Appraisal scale used for included studies  Cochrane RoB 2.0 tool for RCTs  Appraisal rating of included studies  High risk of bias (n=3)  Low risk of bias (n=1)  GRADE completed by systematic review authors  Not reported  Review appraisal score  9 out of 11 in the JBI checklist for systematic reviews and research syntheses | Main findings n=4/31  *Movement behaviour:*  At post-test, sample-weighted improvement in movement behavior was not significant, of small magnitude (d+ = 0.036), and not reliable (i.e. the 95% CIs and PIs crossed zero).  (k=4, d+=0.036, 95% CI (-0.088, 0.161), 95% PI (-0.237, 0.310), p>0.001)  The I^2^ was zero. (I^2^=0.0%)^a^ |
| NICE 2008  Physical activity and children Review 6: Intervention review: adolescent girls  <https://www.nice.org.uk/guidance/ph17/documents/promoting-physical-activity-for-children-consultation-on-the-evidence7> | Publication year of included studies  1997-2006  Review purpose  What interventions are effective in increasing levels of physical activity/core physical skills in adolescent girls aged 11-18 years of age?  Included study designs  RCT (n=2)  Cluster RCT (n=5)  Controlled non-randomised trial (n=4)  Randomised non-controlled trials (n=1)  Outcomes of interest  PA outcomes  -Self-report (validated self-reported assessment only (n=4), self-reported assessment of unknown validity (n=5))  -Objective  (objective assessment only (n=1))  -Combination of objective and validated self-report measures (n=2) | Number of included studies  n=12  Interventions  The 12 studies investigated 13 intervention arms  School-based (single-behaviour) interventions (n=6), including:   - Counselling (n=1), Mediated (n=1), Mediated & Counselling (n=1), Education (n=2), PA self-monitoring (n=1)   School-based (multiple behaviour) interventions (n=5)   - Mediated (n=2), Education (n=2), Education & Mediated (n=1)   Primary healthcare intervention (n=1)   - Mediated & Counselling (n=1)   Home (non-specific setting interventions) (n=1)   - Mediated (n=1)   No information about managing menstruation and the menstrual cycle was reported.  *Focus:*  PA-only (n=7, 7 arms)  PA and at least one other behaviour (e.g. nutrition) (n=5, 6 arms)  *Intervention length:*  Not reported  *Follow-up:*  Immediate post-intervention only (n=2)  Studies with follow-up (n=10) (Ranging from 2 weeks to 4 years)  Participants  Adolescent girls  Girls only (n=6)  Mixed (n=6)  Age  Between 11-13 years (n=8)  Between 14-18 years (n=4)  Countries of interventions  USA (n=6), UK (n=2), Australia (n=1), France (n=1), Belgium (n=1), Ireland (n=1)  Ethnicity  Not reported  Settings  School (including school & community) (n=10)  Primary health care (n=1)  Home (n=1) | Appraisal scale used for included studies  NICE manual  Appraisal rating of included studies  RCTs  High quality (n=2)  Cluster RCT  Moderate quality (n=5)  Controlled non-randomised trial  Poor quality (n=4)  Randomised non-controlled trials  Moderate quality (n=1)  GRADE completed by systematic review authors  Not reported  Review appraisal score  8 out of 11 in the JBI checklist for systematic reviews and research syntheses | Main findings  *School-based (single-behaviour) interventions: (n=6)*  Some school-based interventions, outside of physical education lessons, targeting the single behaviour of physical activity, can lead to moderate-to-large increases in physical activity in adolescent girls for up to 6 months (n=4)  Failed to show an effect (n=2)  *School-based (multiple behaviour) interventions: (n=5)*  Some school-based interventions, outside of physical education lessons, targeting multiple health behaviours, including physical activity, increased physical activity in adolescent girls (n=1)  Failed to show an effect (n=4)  *Primary health care intervention: (n=1)*  Failed to show an effect (n=1)  *Home (non-specific setting interventions): (n=1)*  Mediated interventions can lead to increases in physical activity in adolescent girls (n=1)  Additional findings  *Mediated interventions: (n=6)*  Some mediated interventions lead to increases in physical activity in adolescent girls (n=2)  Interventions delivered via a medium such as computer, phone or printed materials failed to show an effect (n=4)  *Counselling interventions (n=3)*  A counselling intervention can lead to an increase in physical activity in adolescent girls (n=1)  Failed to show an effect (n=2)  *Educational interventions (n=5)*  Educational interventions can increase levels of physical activity in adolescent girls for up to 6 months (n=3)  Failed to show an effect (n=2) |
| Owen et al. 2017  The effectiveness of school-based physical activity interventions for adolescent girls: a systematic review and meta-analysis  <https://doi.org/10.1016/j.ypmed.2017.09.018> | Publication year of included studies  2005-2015  Review purpose  To assess the impact and design of school-based PA interventions targeting adolescent girls.  Included study designs  RCT (n=14) (including: cluster (n=3), pilot (n=1))  Quasi-experimental (n=5)  Case-crossover (n=1)  Outcomes of interest  PA outcomes  -Self-report  -Objective (Accelerometers, Heart Rate, Pedometer) | Number of included studies  n=20  Interventions  Multicomponent (n=10)  Single component interventions (n=10)  No information about managing menstruation and the menstrual cycle was reported.  *Focus:*  Not reported  *Intervention length:*  12-36 months (n=5)  5-11 months (n=5)  <4 months (n=10)  *Follow-up:*  ≥ 12 months (n=2)  ≤ 6 months (n= 8)  Not mentioned (n=10)  Participants  Adolescent schoolgirls  Girls only (n=16)  Mixed (n=4)  Ethnicity  Not reported  Age  Between 11-14 years (n=17)  Between 15-17 years (n=3)  Countries of interventions  USA (n=8), UK (n=4), Australia (n=4), Poland (n=1), Belgium (n=1), Cyprus (n=1), Iran (n=1)  Settings  School (n= 20) | Appraisal scale used for included studies  Modified Risk of Bias Tool (Morton et al. 2016, Pluye et al. 2009)  It uses a 1-4 scoring system: 1= weak, 2= moderate, 3= strong, 4= very strong  A higher risk of bias score indicates better methodological quality, whereas a lower risk of bias score indicates poorer methodological quality.    Appraisal rating of included studies  Very strong risk of bias (n=1)  Strong risk of bias (n=3)  Moderate risk of bias (n=10)  Weak risk of bias (n=6)  GRADE completed by systematic review authors  Not reported  Review appraisal score  8 out of 11 in the JBI checklist for systematic reviews and research syntheses | Main findings  *Meta-Analysis (n=17)*  Twelve studies reported a small effect (g= − 0.29 to 0.26), four studies reported moderate to strong effects (g=0.65 to 1.04)), and one reported a very strong effect size (g=3.43). The meta-analysis revealed a significant small positive treatment effect for school-based PA interventions for adolescent girls (k=17, g=0.37, 95% CI (0.0008, 0.73), p<0.05)  Heterogeneity: There is significant between-study variance (Q = 80.12, p < 0.001; I^2^ = 94.91%).  One intervention was identified as an outlier due to large residual effects (z=7.61). Once this study was removed the average treatment effect was significantly reduced by 0.30, indicating a very small positive effect which approached significance (k=16, g=0.07, 95% CI (−0.002, 0.14), p=0.05).  Heterogeneity was also substantially reduced when the outlier was removed (Q= 23.98, p>0.05; I^2^ = 0.01%).  *Narrative synthesis:*  The three studies excluded from the meta-analysis all indicated positive results.  Additional findings:  *Subgroup analysis*  *Study duration (Short) <6 months*  k=8, g=0.22, 95% CI (−0.06, 0.50), p>0.05  Heterogeneity: Q = 15.01, p<0.05; I^2^= 56.92%  *Study duration (Long) >6 months*  k= 8, g = 0.06, 95% CI (−0.02, 0.14), p>0.05  Heterogeneity: Q = 8.84, p>0.05; I^2^= 0.00%  *Risk of bias */** (weak/moderate)*  k=13, g = 0.09, 95% CI (0.02, 0.17), p<0.05  Heterogeneity: Q = 16.67, p>0.05; I^2^= 0.00%  *Risk of bias ***/**** (strong/very strong)*  k=3, g = 0.01, 95% CI (−0.44, 0.46), p>0.05  Heterogeneity: Q = 4.99, p>0.05; I^2^= 65.95%  *Single component intervention*  k=9, g=0.02, 95% CI (−0.09, 0.14), p>0.05  Heterogeneity: Q = 11.83, p>0.05; I^2^= 0.00%  *Multicomponent intervention*  k=7, g=0.09, 95% CI (0.006, 0.18), p<0.05  Heterogeneity: Q = 11.30, p>0.05; I^2^= 0.02%  *Gender Target (Girls only)*  k=13, g=0.06, 95% CI (−0.02, 0.13), p>0.05  Heterogeneity: Q = 19.35, p>0.05; I^2^= 0.03%  *Gender Target (Mixed)*  k=3, g=0.28, 95% CI (−0.05, 0.61), p>0.05  Heterogeneity: Q = 2.64, p>0.05; I^2^=20.27%.  *Theory included (YES)*  k=12, g=0.07, 95% CI (0.0009, 0.15), p<0.05  Heterogeneity: Q = 18.35, p>0.05; I^2^=0.01%.  *Theory included (NO)*  k=4, g=0.06, 95% CI (−0.33, 0.45), p>0.05  Heterogeneity: Q = 5.38, p>0.05; I^2^=38.75%. |
| Pearson et al. 2015  The effectiveness of interventions to increase physical activity among adolescent girls: a meta-analysis  <https://doi.org/10.1016/j.acap.2014.08.009> | Publication year of included studies  1987-2013  Review purpose  To quantify the effect of physical activity interventions on adolescent girls by including all intervention studies that provided results for girls separately and compared an intervention with a control or non–physical activity comparison  Included study designs  RCTs (n=23)  Controlled trial (n=7)  Quasi-experimental (n=4)  Outcomes of interest  PA behaviour  -Self-report (n=26)  -Objective (n=6)  - Combination of objective and self-report (n=3) | Number of included studies  n=34 studies (independent samples) across 45 reports  Interventions  Educational (n=21)  Environmental (n=4)  Multicomponent (n=9)  *Focus:*  PA only (n=16)  PA and sedentary behaviour (n=4)  PA and diet (n=6)  Heart health (n=4)  Obesity-related (n=4)  *Intervention length:*  ≤ 12 weeks (n=10)  > 12 weeks (n=24)  *Follow-up:*  Immediate post-intervention only (n=22)  Studies with follow-up (n=12) (follow-up period not specified)  Participants  Adolescent girls  Girls only (n=19)  Mixed (data separated for girls) (n=15)  Ethnicity  Not reported  Age  Between 12-18 years  Older adolescents with mean age > 16 years (n=12)  Younger adolescents with mean age < 16 (n=22)  Countries of interventions  USA (n=21), Poland (n=2), Canada (n=1), The Netherlands (n=1), Australia (n=3), UK (n=3), Belgium (n=1), France (n=1), Iran (n=1)  Settings  Community (n=1)  Community and parent (n=1)  School (n=19)  School and outside of school (n=13) | Appraisal scale used for included studies  Cochrane RoB tool for RCTs  Appraisal rating of included studies  High (n=4)  Moderate (n=12)  Low (n=18)  GRADE completed by systematic review authors  Not reported  Review appraisal score  7 out of 11 in the JBI checklist for systematic reviews and research syntheses | Main findings  The overall effect size was small (k=35^b^, g=0.350, 95% (0.12, 0.58), p <0.001), but significant and indicated that physical activity behavior change is possible but likely to be challenging.  Heterogeneity: (Q_T_=1436.90, τ^2^=0.421, I^2^=98%)  Additional findings  Subgroup analyses showed greater effects for interventions that were theory based, performed in schools. Where girls only, with younger girls, used multicomponent strategies, and involved targeting both physical activity and sedentary behaviour.  Subgroup analysis  *Sample characteristics (girls only vs mixed)*  Girls only (k=19, g= 0.439, 95% CI (0.162, 0.717), p<0.01) Heterogeneity (τ^2^=0.751, I^2^=98.04)  Mixed (k=15, g=0.239, 95% CI (-0.076, 0.553), p>0.01) Heterogeneity (τ^2^=0.074, I^2^=90.08)  *Intervention type*  Educational interventions (k=21, g=0.225, 95% CI (-0.060, 0.509), p>0.01) Heterogeneity (τ^2^=0.105, I^2^=89.21)  Environmental (k=4, g=0.372, 95% CI (-0.301, 1.046), p>0.01) Heterogeneity (τ^2^=0.130, I^2^=71.60)  Multicomponent (k=9, g=0.618, 95% CI (0.197, 1.039), p<0.01) Heterogeneity (τ^2^=0.827, I^2^=99.18)  *Intervention length*  ≤ 12 weeks (k=10, g=0.384, 95% CI (-0.044, 0.812), p>0.01) Heterogeneity (τ^2^=0.201, I^2^=89.95)  > 12 weeks (k=24, g=0.336, 95% CI (0.063, 0.610), p<0.01) Heterogeneity (τ^2^=0.472, I^2^=98.28)  *Study quality*  High (k=4, g=0.524, 95% CI (-0.166, 1.214), p>0.01) Heterogeneity (τ^2^=1.104, I^2^=98.74)  Low (k=18, g=0.261, 95% CI (-0.070, 0.592), p>0.01) Heterogeneity (τ^2^=0.081, I^2^=79.76)  Moderate (k=12, g=0.420, 95% CI (0.020, 0.820), p<0.01) Heterogeneity (τ^2^=0.577, I^2^=98.98)  *Theoretical approach (within subgroup differences)*  Atheoretical (k=10, g=0.180, 95% CI (-0.241, 0.601), p>0.01) Heterogeneity (τ^2^=0.032, I^2^=59.64)  Theoretical (k=24, g= 0.422, 95% CI (0.148, 0.696), p<0.01) Heterogeneity (τ^2^=0.494, I^2^=98.35) |
| Reed et al. 2017  Impact of workplace physical activity interventions on physical activity and cardiometabolic health among working-age women: a systematic review and meta-analysis  <https://doi.org/10.1161/CIRCOUTCOMES.116.003516> | Publication year of included studies  1985 to 2014  Review purpose  To examine the effectiveness of workplace interventions for increasing MVPA levels among working-age women in such settings  Included study designs  RCTs (n=19)  Randomised trials (n=2)  Quasi-experimental trial (n=1)  Pre/post trials (n=2)  Outcomes of interest  PA outcomes:  MPVA  -Self-report  -Objective  MVPA was defined as activity involving an energy expenditure of ≥3 metabolic equivalents (METs), ≥40% of VO_2_ reserve, ≥46% VO_2_ peak, ≥64% of peak heart rate, ≥12 rating of perceived exertion, or >100 steps per minute.  MET minutes per week  -Self-report  METs per week  -Self-report | Number of included studies  n=24  Interventions  Single intervention strategy (n=3)  Multiple intervention strategies (n=21)  No information about managing menstruation and the menstrual cycle was reported.  *Intervention length:*  Range 15 days to 2 years  < 3 months/12 weeks (n=7)  3 months/12 weeks < 6 months (n=9)  6 months (n=6)  >6 months (n=2)  *Follow-up:*  Not reported  Participants  Working-age women (>80% of sample)  Age  Between ages 17-51 (n=20, 83%)  Over the age of 51 (n=4, 17%)  Ethnicity  Not reported  Countries of interventions  USA (n=12), Canada (n=1), Canada and USA (n=1), UK (n=2), Australia (n=2), Finland (n=2), The Netherlands (n=2),  Ireland (n=1), Sweden (n=1)  Settings  Work settings | Appraisal scale used for included studies  Cochrane RoB tool for RCTs  Appraisal rating of included studies  The largest risk of bias emanated from the use of incomplete outcome data (because of high [>10%] attrition), selective reporting (because of the high number of studies with unpublished women-specific data provided for this review), and other bias (self-report measures).  GRADE completed by systematic review authors  RCTs: downgraded from high to very low due to high risk of bias (ie, >50% of included studies had high attrition bias, selective reporting, and used self-report measures); high heterogeneity for minutes per week of MVPA, METs per week, and MET minutes per week (I^2^=86% - 97%, p<0.0001); and evidence of publication bias (ie, asymmetrical funnel plot)  Non-RCT : downgraded from moderate to very low because of a high risk of bias (ie, >50% of included studies had high attrition bias, selective reporting, and did not blind participants)  Review appraisal score  11 out of 11 in the JBI checklist for systematic reviews and research syntheses | Main findings  *Minutes per week of MVPA (n=12)*  Results of the meta-analyses reveal that  the interventions did not significantly increase minutes per week of MVPA (SMD=0.38; 95% CI, (−0.15, 0.92), p=0.16)  Heterogeneity: (I^2^=97%, p<0.00001)  *METs per week (n=3)*  The interventions did not significantly increase METs per week (SMD=0.11; 95% CI (−0.48, 0.71), p=0.71)  Heterogeneity: (I^2^=86%, p<0.00001)  *MET min/week (n=4)*  The interventions significantly increased MET minutes per week (SMD=2.07, 95% CI (1.44, 2.69), p<0.00001) equating to a MD of ≈210 MET minutes per week.  Heterogeneity: (I^2^=97%, p<0.00001) |
| Voskuil et al. 2017  Effect of physical activity interventions for girls on objectively measured outcomes: a systematic review of randomized controlled trials  <http://dx.doi.org/10.1016/j.pedhc.2016.03.003> | Publication year of included studies  1985-2014  Review purpose  To evaluate the effect of PA interventions on accelerometer-measured PA of girls (elementary through high school) participating in randomized controlled trials  Included study designs  RCT (n=15) (including:  group RCTs (n=7))  Outcomes of interest  PA outcomes:  -Objective (e.g.: accelerometer measured PA calculating MET-weighted minutes of MVPA, and total MVPA) (n=5) | Number of included studies  n=15 (out of 15 included studies only 5 reported on PA outcomes and are extracted below)  Interventions  Multicomponent (n=5)  No information about managing menstruation and the menstrual cycle was reported.  *Focus:*  Not reported  *Intervention length:*  Ranging between 16 weeks to 2 years  <11 months (n=1)  >11 months (n= 4)  *Follow-up:*  Immediate post-intervention only (n=3)  ≤12 months (n=2)  Participants  Adolescent school girls  Age  Between 8-18 years  Ethnicity  African American (n=2)  Primarily Caucasian (n=1)  Latina (n=1)  Diverse sample (n=1)  Countries of interventions  USA (n=4)  Australia (n=1)  Settings  School based (n=1)  Community based (n=2)  Community and home based (n=1)  Community and school based (n=1) | Appraisal scale used for included studies  Cochrane Collaboration's tool was used, with these categories used as criteria: selection, performance, detection, attrition, and reporting biases. The CASP guidelines and recommendations from the Effective Practice and [Organization of Care](https://www.sciencedirect.com/topics/nursing-and-health-professions/organization-of-care) group were also used to guide the appraisal.  Quality appraisal was conducted using the qualitative descriptors high, low, or unclear  Appraisal rating of included studies  Low risk of bias in 6 out of 6 domains (n= 2)  Low risk of bias in 5 out of 6 domains (n=1)  Low Risk of bias in 4 out of 6 domains (n= 2)  GRADE completed by systematic review authors  Not reported  Review appraisal score  7 out of 11 in the JBI checklist for systematic reviews and research syntheses | Main findings  *PA outcomes*  Of the five studies measuring PA objectively, **only one study had statistically significant increases** in accelerometer-measured PA for girls in intervention groups compared with control groups. Modest increases in MET-weighted minutes of MVPA (10.9 MET-weighted minutes, *p*=0.03; 95% CI (0.5, 21.2)) and total MVPA (1.6 mean minutes/day, *p*=0.049; 95% CI (0.0, 3.0)) were reported. These differences were noted only in the third and final year of the intervention, when it was directed by program champions, compared with the study staff, who led the intervention in the first 2 years. No statistically significant differences were found for total PA in this study. |

**Key:** CASP - Critical Appraisal Skills Programme; CEBM – Centre for Evidence-based Medicine; CI - confidence interval; ECPAP - extra-curricular physical activity programs; GRADE - Grading of Recommendations, Assessment, Development, and Evaluations; HCP - healthcare professionals; ITT – intention-to-treat; MET - Metabolic Equivalent of Task; MVPA – Moderate- to vigorous-physical activity; NICE - The National Institute for Health and Care Excellence; PA - physical activity; PE - physical education; PI – prediction interval; RCT - randomised controlled trial; RoB - risk of bias; ROBINS-I - Risk Of Bias In Non-randomised Studies - of Interventions; SMD - standardised mean difference; SRM - Supporting role model; k - number of effect sizes; g = effect size (Hedges' g); d+ = sample weighted average effect size

^a^ Tufanaru et al. 2015 do not recommend meta-analysis (random effects model) on a sample smaller than 5.

With a small number of studies (< 20), the I^2^ confidence interval should be interpreted very cautiously (Huedo-Medina et al 2006).

^b^ Forest plot shows 35 instead of 34 independent samples

**References for appraisal scales used in the included reviews:**

Olivo SA, Macedo LG, Gadotti IC, Fuentes J, Stanton T, Magee DJ: **Scales to assess the quality of randomized controlled trials: a systematic review**. *Physical Therapy* 2008, **88**(2):156-175.

Hooper P, Jutai JW, Strong G, Russell-Minda E: **Age-related macular degeneration and low-vision rehabilitation: a systematic review**. *Canadian Journal of Ophthalmology* 2008, **43**(2):180-187.

Morton KL, Atkin AJ, Corder K, Suhrcke M, van Sluijs EM: **The school environment and adolescent physical activity and sedentary behaviour: a mixed-studies systematic review**. *Obesity Reviews* 2016, **17**(2):142-158.

Pluye P, Gagnon MP, Griffiths F, Johnson-Lafleur J: **A scoring system for appraising mixed methods research, and concomitantly appraising qualitative, quantitative and mixed methods primary studies in Mixed Studies Reviews**. *International Journal of Nursing Studies* 2009, **46**(4):529-
