# Supplementary material for: Interventions that support women, girls, and people who menstruate to participate in physical activity: a rapid overview of reviews
Source: BMC Public Health. 2026 Mar 27;26:1472. doi: 10.1186/s12889-026-27122-9 (PMC13147802; doi:10.1186/s12889-026-27122-9)
Supplement: Supplementary file 7 — Additional file 7: Table of interventions and behaviour change theories A table containing components and underpinning behaviour change theories of interventions included in the systematic reviews. [file 12889_2026_27122_MOESM7_ESM.docx]

**Additional file 7: Table of interventions and behaviour change theories**

| **Citation**  **Number of included studies**  **Participants**  **Age** | **Type of interventions: definition/description** | **Behaviour change theories used in interventions** |
| --- | --- | --- |
| **Young and adolescents girls** | | |
| Allison et al. 2017  n=4    Girls  11–25 | Interventions  Partnership working (n=1): community sports clubs and national governing bodies. This involved forging relationships to ensure  progressive and coherent pathways, which was seen as crucial for retention in team sport  Grant funding (n=1)  Multicomponent (n=2)  List of intervention components  Training for staff, action plan, grant funding, partnership working, resource provision, how-to guide, gender-specific research insights, merchandise | Behaviour change theories  Not reported |
| Biddle et al. 2014a, b  n=22  Preadolescents  5-12 | Interventions  Educational (n=9): not defined  Environmental (n=4): not defined  Multicomponent (n=9): educational plus environmental change  List of intervention components  After-school programme; family involvement; game equipment provided for class; classroom curriculum; behaviour modification lessons; specialist led PE; self-management sessions; redesigning playground environment; PE class with added daily 15 minute exercise sessions; health and nutrition component; allocation of school courts; skill building; self-monitoring; daily step target; interactive animated lessons; community awareness; health education; increased PE lessons; summer camp | Behaviour change theories  While 12 studies were described as having a behaviour change theory foundation, no description of the theories were reported. |
| Camacho-Minano et al. 2011  n=21  Young and adolescent girls  5-18 | Interventions  Educational and multicomponent but interventions are not formally grouped. Main grouping is based on the setting, e.g.: school, community, primary care  List of intervention components  School-based after-school programmes; PE specific interventions; PE with school environmental and policy change; girl scout troops; summer camp programme; family involvement; school-community links | Behaviour change theories  14 studies reported using at least one behaviour change theory. These were:  Social cognitive theory (n=8);  Transtheoretical model of behaviour (n=3);  Self-determination theory (n=1);  Theory of meaning of behaviour (n=1);  Health promotion model (n=2);  Social action theory (n=1);  Social ecological model (n=2) |
| Houle et al. 2020  n=17 (out of 17 included studies only 4 reported on PA outcomes and are extracted below)  Adolescent girls  11 – 17 | Interventions  Extra-curricular physical activity programs: not defined  List of intervention components  PA programme (n=2)  -Dance Programme (n=2)  Multi-approach programme (n=2)  -All included some discussion either one-to one or groups (health, PA, nutrition, empowerment and skill development)  -Some included a motivational component  -Monthly newsletters  -Text messages | Behaviour change theories  Not reported |
| Kelly et al. 2024  Peer reviewed paper (n=1)  Grey literature (n=15)  Peer reviewed paper: Adolescents  11-16  Grey literature: Not reported | Interventions  Peer reviewed paper: an elite sports role model visited the schools  Grey literature: Sport role model encounters. One off encounters (n=10), multiple encounters (n=5, 2 with minimal interaction)  List of intervention components  Peer reviewed paper: “Sky Living for Sports”: All schools were funded for between 2 and 3 months for 3 years to complete a variety of novel (e.g. skiing, judo, skateboarding, and orienteering) activities within school time  “Changing Lives” (Intervention): In addition to “Sky Living for Sport”, an elite sports role model visited the schools once per year. Sporting Role Models were world-class elite athletes selected based on capability to interact with adolescents by sharing their own struggles with adversity and how they overcame them  Grey literature: Sporting Role Models: Olympians/ Paralympians (n=5); Intercounty Gaelic Games players (n=3), High-profile, elite, or professional athletes (n=7). Most included studies did not include information on SRM matching procedures | Behaviour change theories  Peer reviewed paper: No theoretical framework was visibly integrated into the program design  Grey literature: Overall, there was a scarcity of information on program design in relation to evidence-informed approaches, theoretical frameworks, specifying program aims, and outlining evaluation frameworks or processes |
| NICE 2008  n=12  Adolescent girls  11-18 | Interventions  School-based (single-behaviour) interventions (n=6), including:  -Counselling (n=1), Mediated (n=1), Mediated & Counselling (n=1), Education (n=2), PA self-monitoring (n=1)  School-based (multiple behaviour) interventions (n=5)  -Mediated (n=2), Education (n=2), Education & Mediated (n=1)  Primary healthcare intervention (n=1)  -Mediated & Counselling (n=1)  Home (non-specific setting interventions) (n=1)  -Mediated (n=1)  Mediated interventions: delivered via a medium such as computer, phone or printed materials  List of intervention components  Activity sessions with and without qualified teacher; Psychological (knowledge, attitudes, beliefs and motivation), social (social support), and environmental (physical, structural and institutional) factors were targeted; tailored feedback; Support materials were also provided to parents; promoting physical activity; self-monitoring techniques, stage-matched counselling, teacher-led extra-curricula physical activity, and multi-level programming targeting psychological, social and environmental correlates; education, mediated approaches, and broad-based education and policy initiatives; educational material | Behaviour change theories  Transtheoretical Model (n=1)  Not reported (n=11) |
| Owen et al. 2017  n=20  Adolescent schoolgirls  11-17 | Interventions  Multicomponent (n=10): not defined  Single component interventions (n=10): not defined  List of intervention components  School environment adaptions; modified PE lessons; extra-curricular PA sessions; educational sessions; counselling sessions; further opportunities to be physically active (e.g., lunch and break time PA clubs)    Single-component interventions: modified PE lessons; after-school dance interventions; educational-based interventions; playground intervention | Behaviour change theories  13 studies reported using at least one behaviour change theory, and these were:  Social Cognitive Theory (n=4);  The Theory of Planned Behaviour and The Trans-theoretical Model;  Self-determination theory (n=3);  Social Cognitive Theory and the Trans-Theoretical Model;  Pender’s Health Promotion Model (n=3);  The Trans-theoretical Model (n=2);  The Theory of Meanings of Behaviour  Operant Learning Theory;  Organizational Change Theory;  The Diffusion of Innovation Model in a Social-ecologic Framework;  Social Action Theory  1 study used Hellison’s Model of Teaching Responsibility through PA  6 studies did not specify the use of behaviour change theory. |
| Pearson et al. 2015  n=34 studies (independent samples) across 45 reports  Adolescent girls  12-18 | Interventions  Educational (n=21): not defined  Environmental (n=4): not defined  Multicomponent (n=9): included strategies that targeted dietary, physical activity, and sedentary behaviours using a number of  methods to target and change unhealthy patterns. Methods used to decrease undesirable behaviours contained combinations of **support components** (ie, family, friends, etc.), **individual components** (ie. specifically tailored programs for individuals/groups), **choice components** (different options to facilitate behaviour change), and **educational and environmental components** targeting during and after school behaviours.  List of intervention components  Class activities (yoga, aerobics, swimming, weight-training, dance etc.);  lectures on time management; body image; motivation; nutrition; strength training;  Taught by teachers, modules on (physical education, diet, smoking, stress, problem solving). Female only PE classes discussion;  Classroom based focus on changing environmental; personality, and behavioural attributes (including videotaped instructions and goal setting, self-monitoring, social support, and how to change environment).  Encouraging additional changes to PE classes; school cafeteria; increased supervision; equipment, and activities.  Health and nutrition; increasing physical activity; addressing issues associated with obesity (e.g. depression, disordered eating patterns, poor body image); training participants’ primary care providers to support behavioural weight management; Parental group sessions;  Providing physical and health education;  Creating supportive school environment, school health services, staff health promotion, and family based and community-based activities Partnership with families and community groups increasing (knowledge, attitudes, beliefs, and motivation for physical activity); social support from parents, peers, teachers, and physical activity instructors.  Individual Counselling session. | Behaviour change theories  24 studies were reported as having a behaviour change theories however, not defined or specified as to which study.  The theories included:  Social cognitive theory;  The transtheoretical model;  Behavioral determinants;  Behavioral choice;  Health promotion model,  Social learning theory |
| Voskuil et al. 2017  n=15 (out of 15 included studies only 5 reported on PA outcomes and are extracted below)  Adolescent school girls  8-12 | Interventions  Multicomponent (n=5): not defined  List of intervention components  Circuit training, cardiovascular activity, strength training, motivational interviewing; Enhanced school sport sessions, interactive seminars, nutrition workshops, lunchtime PA sessions, parent newsletters, handbooks and pedometers for self-monitoring, text messaging for social support; group behavioural counselling; after-school dance sessions, home/family-based programme to reduce screen use; health education, PE class, school/community partners planned programmes, programme champions | Behaviour change theories  Social ecological theory and other theories (n=1) |
| **Adult women** | | |
| Amiri Farahani et al. 2015  n=9  Women  18-65 | Interventions type  Multicomponent (n=9): not defined  List of intervention components  Social support; goal setting; self-monitoring; verbal encouragement and written reinforcement to achieve short-term and long-term PA goals; cultural facilitators and expert consultants for teaching behavioural strategies and skills to help the women implement an individualised health plan; contact information for local healthy PA resources; problem-solving training for overcoming the barriers of PA; media messages; economic incentive (free gym membership) | Behaviour change theories  All nine studies reported using at least one behaviour change theory. These were:  Social cognitive theory (n=7);  Social cognitive theory and social marketing theory (n=1);  Social ecological model (n=1) |
| Madden et al. 2020  n=20 studies across 23 reports  Working women  33.2±7.8 and 48.77+9.27 | Interventions  Exercise (n=5)  Interrupted sitting (n=1)  Multicomponent (n=14)  List of intervention components  Education, goal-setting, exercise, peer support, incentives, self-monitoring, counselling, health assessment, self-efficacy, weigh-in sessions, cognitive restructuring, environment scan, feedback, goal monitoring, motivational interviewing, overcoming barriers, problem solving, relapse prevention, skill building | Behaviour change theories  Social ecological model, self-efficacy theory, transtheoretical model of health behaviour change, social cognitive theory (or social learning theory), health belief model, self-determination theory (n=8) |
| Reed et al. 2017  n=24  Working-Age Women  17-51 (83%) | Interventions  Workplace interventions  Single intervention strategy (n=3): not defined  Multi intervention strategy (n=21): not defined  List of intervention components  Counselling, appointments with occupational nurses or Registered Dieticians, fitness testing, messages/emails providing feedback on PA, diet, or health promotion, stages of change–based interventions, personal, partner, or team goal-setting, self-monitoring of body mass or PA using activity monitors or questionnaires, team or individual competitions, online social networks to connect with friends and make public postings, tailored web-based PA advice, incentives, PA prescriptions, knowledge quizzes, educational materials (eg, leaflets, posters, pamphlets, newsletters, reminders, or fridge magnets), educational sessions, expressions of management support, active workstations, promotion of incidental office activity, pedometer challenges, encouraged social support, enhanced self-efficacy and awareness of benefits of PA, reduction of perceived barriers to PA, reduced work hours, access to a motivational website, tracking and simulation tools, personalized diet guidelines, online discussion forums, or weekly weigh-in sessions (online or in-person). self-monitoring of PA using an activity monitor or monthly calendar or tailored emails | Behaviour change theories  Not reported |
| **Mixed (both young and adolescent girls and adult women)** | | |
| Matheson et al. 2023  n=31 (out of 31 included studies only 4 reported on Movement behaviour outcomes and are extracted below)  Girls and women  0-17  >35 | Interventions  Body image or movement-based interventions  Unimodal (n=1)  Multimodal (n=3)  List of intervention components  Unimodal: hatha yoga’  Mulitmodal, including: physical activity intervention, Healthy me (strengths-based approach to enhance positive body image), Healthy body image program | Behaviour change theories  Self-Determination Theory (n=1)  Exercise and Self-esteem model (n=1)  Social Cognitive Theory (n=1)  Embodiment Theory / Media Literacy (n=1) |
| **Mothers and daughters** | | |
| Barnes et al. 2018  n=14 studies across 16 reports  Mothers and daughters  8-19 (daughters)  32-45.2 (mothers mean age) | Interventions  Community-based intervention for mothers and daughters that targeted physical activity, fitness, nutrition, or adiposity  List of intervention components  Community-based tutoring, after-school, summer camp, and family- and home-based programs | Behaviour change theories  Not reported |
| Brennan et al. 2021  n=11  Mothers and daughters  7-17 (daughters)  28-50 (mothers) | Interventions  Described as mother and daughter interventions (no further detail reported)  List of intervention components  Nine studies described the materials used in the interventions, involving newsletters, booklets, certificates, DVDs, stickers, jump ropes, balls, weights, pedometers, and log books  All studies, except one reported the procedures involved in the physical activity component of the interventions such as, dance, fitness, interactive games and activities and group walking | Behaviour change theories  Eight studies specified a named theory. These were:  Social cognitive theory (n = 5)  Behavior change wheel for intervention design (n=1)  Social cognitive theory with family systems theory (n=2)  Social cognitive theory with social-ecological model (n=1)  Of these eight studies, only four measured relevant theoretical constructs |
